# Modeling and Analysis of the Two-Dimensional Axisymmetric Acoustofluidic Fields in the Probe-Type and Substrate-Type Ultrasonic Micro/Nano Manipulation Systems

**DOI:** 10.3390/mi11010022

**Published:** 2019-12-24

**Authors:** Pengzhan Liu, Qiang Tang, Songfei Su, Jie Hu, Yang Yu

**Affiliations:** 1 State Key Lab of Mechanics and Control of Mechanical Structures, Nanjing University of Aeronautics and Astronautics, Nanjing 210016, China; 2 Department of Mechanical Engineering and Materials Science, Duke University, Durham, NC 27708, USA; 3 Faculty of Mechanical and Material Engineering, Huaiyin Institute of Technology, Huaian 223003, China; tangqiang102@126.com; 4 School of Mechanical Engineering, Nanjing Institute of Technology, Nanjing 211167, China; susongfeinh@163.com; 5 School of Engineering, Jiangxi Agricultural University, Nanchang 330045, China; hujie9@nuaa.edu.cn; 6 School of Civil and Environmental Engineering, University of Technology Sydney, Sydney, NSW 2007, Australia

**Keywords:** acoustic streaming, acoustofluidics, micro/nano, manipulation, FEM

## Abstract

The probe-type and substrate-type ultrasonic micro/nano manipulation systems have proven to be two kinds of powerful tools for manipulating micro/nanoscale materials. Numerical simulations of acoustofluidic fields in these two kinds of systems can not only be used to explain and analyze the physical mechanisms of experimental phenomena, but also provide guidelines for optimization of device parameters and working conditions. However, in-depth quantitative study and analysis of acoustofluidic fields in the two ultrasonic micro/nano manipulation systems have scarcely been reported. In this paper, based on the finite element method (FEM), we numerically investigated the two-dimensional (2D) axisymmetric acoustofluidic fields in the probe-type and substrate-type ultrasonic micro/nano manipulation systems by the perturbation method (PM) and Reynolds stress method (RSM), respectively. Through comparing the simulation results computed by the two methods and the experimental verifications, the feasibility and reasonability of the two methods in simulating the acoustofluidic fields in these two ultrasonic micro/nano manipulation systems have been validated. Moreover, the effects of device parameters and working conditions on the acoustofluidic fields are clarified by the simulation results and qualitatively verified by the experiments.

## 1. Introduction

In the past two decades, acoustic micro/nano manipulation methods [1,2,3] have shown tremendous potential applications in biomedical engineering [4,5], bioanalytical chemistry [6,7], material science [8,9], micro/nano fabrication [10,11], lab-on-a-chip (LOC) technology [12,13] and so forth. Compared to other physical micro/nano manipulation methods, such as optical methods [14,15], magnetic methods [16,17,18], mechanical methods [19,20], and dielectrophoretic (DEP) methods [21,22,23], acoustic micro/nano manipulation methods possess the merits such as simple fabrication, little selectivity to optical/electrical/magnetic properties of manipulated samples, and good compatibility with other LOC components [1,2]. The acoustic micro/nano manipulation devices can be divided into two major categories, i.e., surface acoustic wave (SAW)-based devices [24,25] and bulk acoustic wave (BAW)-based devices [1]. For SAW-based devices, diverse manipulation functions, such as trapping [26,27], patterning [28,29], separation [30,31], concentration [32,33], mixing [34,35], and rotation [36,37] have been implemented by various groups. For BAW-based devices, manipulation functions such as levitation [38,39], assembly [40,41], focusing [42,43] and sorting [44] have also been realized by many researchers.

It is well known that in acoustic micro/nano manipulation systems, acoustic radiation force [45] and acoustic streaming [46,47,48,49,50] are employed to implement micro/nano manipulation functions. Therefore, both experimental measurement and numerical modeling of these two kinds of acoustic physical effects are essential for experimental phenomenon explanations and parameter optimizations [51,52,53,54,55,56]. For acoustic radiation force, numerical simulations based on Gor’kov’s theory [45] have grown to be relatively mature [57,58]. For acoustic streaming, to date, there have been three mainstream simulation methods, that is, the limiting/slip velocity method [59,60,61], the perturbation method (PM) [46,62,63,64,65,66], and the Reynolds stress method (RSM) [47,67]. The limiting/slip velocity method can only be applied to simulate acoustic streaming driven by boundary layers [68], but is inapplicable for simulating Eckart streaming [69]. The PM and RSM are capable of simulating most kinds of acoustic streaming fields, including Eckart streaming [69], Rayleigh streaming [70] and Schlichting streaming [71]. Commonly, numerical simulations of 2D acoustic streaming fields are restricted to 2D rectangular coordinate system-based simulations by assuming that longitudinal lengths of microchannels [72,73] or depths of chambers [74] are infinite. However, numerical simulations of 2D axisymmetric acoustic streaming fields have rarely been reported, which may play important roles in droplet-based acoustofluidics [66,67].

Although both the PM and RSM can be applied to simulate most kinds of acoustic streaming fields, in the reported literatures in the field of Acoustofluidics, the PM is more inclined to be utilized for simulating acoustic streaming fields in SAW acoustofluidic devices and systems [32,61,72], while the RSM is more adopted to simulate acoustic streaming fields in BAW acoustofluidic devices and systems [1,67,74]. However, there has been no literature with respect to comparisons of simulation results computed by the PM and RSM, and some researchers even get confused when facing the issue that how to select the most appropriate method for simulating acoustic streaming fields in specific acoustofluidic systems. Therefore, it is necessary to physically understand the algorithm difference between the PM and RSM for engineering simulations of acoustic streaming.

The authors’ group has proposed many acoustic micro/nano manipulation principles [1] based on the probe-type [75,76] and substrate-type [77,78] ultrasonic devices. For the probe-type ultrasonic devices, the ultrasonic probe-droplet-substrate system, in which an ultrasonically vibrating micro manipulating probe (MMP) is inserted into a droplet of micro/nanoscale object suspension on a stationary substrate, is established to manipulate micro/nanoscale objects at the droplet-substrate interface near the MMP. Based on the ultrasonic probe-droplet-substrate system, manipulation functions such as trapping [75], rotation [79], removal [80] and concentration [81] have been implemented. For the substrate-type ultrasonic devices, the droplet-ultrasonic substrate system, in which a droplet of micro/nanoscale object suspension is placed on an ultrasonically vibrating substrate, is established to manipulate micro/nanoscale objects at the droplet-substrate interface. Concentration of silver nanowires (AgNWs) has been realized by this system [82]. However, quantitative analysis of the acoustofluidic fields (especially the acoustic streaming fields) in the aforementioned systems is very insufficient, which hinders the deeper understanding of dynamics of micro/nanoscale objects in the manipulation processes, as well as the optimization of devices’ parameters.

In this work, based on the finite element method (FEM), we quantitatively investigate the 2D axisymmetric acoustofluidic fields in the probe-type and substrate-type ultrasonic micro/nano manipulation systems by the PM and RSM, respectively. By numerical simulations and experimental verifications, the effects of device parameters and working conditions on the acoustofluidic fields have been elucidated by the computation and verified by the experiments.

## 2. Basic Theories and Simulation Methods of 2D Axisymmetric Acoustic Streaming Fields

The fundamental governing equations of acoustic streaming theory of the perturbation method (PM) and Reynolds stress method (RSM) are firstly introduced, respectively, and then the two simulation methods are elaborated.

### 2.1. The Perturbation Method (PM)

Herein, the fluid is assumed to be homogeneous and isotropic, in which the continuity and the compressible Navier-Stokes (N-S) equations are satisfied as

(1) $$\frac{\partial \rho }{\partial t}+\nabla \cdot (\rho u)=0\;$$

(2) $$\rho (\frac{\partial u}{\partial t}+u\cdot \nabla u)=-\nabla p+\mu \nabla^{2}u+(\mu_{b}+\frac{1}{3}\mu )\nabla (\nabla \cdot u)$$

where *ρ*, *μ*, and *μ_b_* are the mass density, shear viscosity, and bulk viscosity of the fluid, respectively; *p* and ***u*** are the pressure and velocity (Eulerian) in the fluid, respectively. 

It is assumed by Nyborg’s perturbation theory [46] that the second-order acoustic streaming field is superposed on the first-order acoustic field. Using this theory, the fluid density, pressure, and velocity (absence of background flow) can, respectively, be expressed as

(3) $$\rho =\rho_{0}+\rho_{1}+\rho_{2}+\cdots$$

(4) $$p=p_{0}+p_{1}+p_{2}+\cdots$$

(5) $$u=u_{1}+u_{2}+\cdots$$

where the subscripts 0, 1 and 2 represent the static (absence of sound), first-order and second-order quantities, respectively. By substituting Equations (3)–(5) into Equations (1) and (2) and only considering the equations of the first-order terms, equations for solving the first-order acoustic field can be obtained as

(6) $$\frac{\partial \rho_{1}}{\partial t}+\rho_{0}\nabla \cdot (u_{1})=0$$

(7) $$\rho_{0}\frac{\partial u_{1}}{\partial t}=-\nabla p_{1}+\mu \nabla^{2}u_{1}+(\mu_{b}+\frac{1}{3}\mu )\nabla (\nabla \cdot u_{1})$$

Repeating the above process, considering the equations of the second-order terms and taking the time average, the continuity and N-S equations for solving the second-order time-averaged acoustic streaming field can be expressed as

(8) $$\langle \frac{\partial \rho_{2}}{\partial t}\rangle +\rho_{0}\nabla \cdot \langle u_{2}\rangle +\nabla \cdot \langle \rho_{1}u_{1}\rangle =0$$

(9) $$\rho_{0}\langle \frac{\partial u_{2}}{\partial t}\rangle +\rho_{0}\langle u_{1}\cdot \nabla u_{1}\rangle +\langle \rho_{1}\frac{\partial u_{1}}{\partial t}\rangle =-\nabla \langle p_{2}\rangle +\mu \nabla^{2}\langle u_{2}\rangle +(\mu_{b}+\frac{1}{3}\mu )\nabla (\nabla \cdot \langle u_{2}\rangle )$$

where 〈*A*〉 denotes the time average of the quantity *A* over a full oscillation time period. For the steady second-order acoustic streaming flow state, $\langle \frac{\partial \rho_{2}}{\partial t}\rangle =0$ and $\langle \frac{\partial u_{2}}{\partial t}\rangle =0$, and then Equations (8) and (9) become

(10) $$\rho_{0}\nabla \cdot \langle u_{2}\rangle =-\nabla \cdot \langle \rho_{1}u_{1}\rangle$$

(11) $$-\nabla \langle p_{2}\rangle +\mu \nabla^{2}\langle u_{2}\rangle +(\mu_{b}+\frac{1}{3}\mu )\nabla (\nabla \cdot \langle u_{2}\rangle )=\rho_{0}\langle u_{1}\cdot \nabla u_{1}\rangle +\langle \rho_{1}\frac{\partial u_{1}}{\partial t}\rangle$$

It is seen that the physical quantities of the first-order acoustic field act as the source terms (at the right-hand sides of Equations (10) and (11)) for the second-order acoustic streaming field (at the left-hand sides of Equations (10) and (11)). Thus, the driving force of acoustic streaming ***F_P_*** can be written as

(12) $$F_{P}=-\langle \rho_{1}\frac{\partial u_{1}}{\partial t}\rangle -\rho_{0}\langle u_{1}\cdot \nabla u_{1}\rangle$$

### 2.2. The Reynolds Stress Method (RSM) 

It is claimed by Lighthill that acoustic streaming is forced by the action of the Reynolds stress, which is defined as the mean value of the acoustic momentum flux [83]. In acoustic fields, the second-order tensor form of the Reynolds stress can be expressed as

(13) $$R=\langle \rho_{0}u_{1}u_{1}\rangle$$

The divergence of the Reynolds stress tensor can cause a net force per unit volume, i.e., the driving force of acoustic streaming ***F_R_***, to act on the fluid, and it can be written as

(14) $$F_{R}=-\nabla \cdot R=-\nabla \cdot \langle \rho_{0}u_{1}u_{1}\rangle =-\rho_{0}\langle \left(u_{1}\cdot \nabla )u_{1}+u_{1}(\nabla \cdot u_{1}\right)\rangle$$

The steady acoustic streaming field generated by ***F_R_*** satisfies the N-S equation

(15) $$\rho_{0}(u_{2}\cdot \nabla u_{2})=-\nabla \langle p_{2}\rangle +\mu \nabla^{2}\langle u_{2}\rangle +(\mu_{b}+\frac{1}{3}\mu )\nabla (\nabla \cdot \langle u_{2}\rangle )+F_{R}$$

The acoustic streaming field also satisfies the continuity equation

(16) $$\nabla \cdot \langle u_{2}\rangle =0$$

According to the algorithm of the divergence of the second-order tensor in the 3D cylindrical coordinate system (*r*, *θ*, *z*), the *r*-, *θ*- and *z*-directional components of ***F_R_*** can be expressed as

(17) $$F_{r}=-\frac{\partial \langle \rho_{0}u_{1r}{}^{2}\rangle }{\partial r}-\frac{1}{r}\frac{\partial \langle \rho_{0}u_{1r}u_{1\theta }\rangle }{\partial \theta }-\frac{\partial \langle \rho_{0}u_{1r}u_{1z}\rangle }{\partial z}-\frac{\langle \rho_{0}u_{1r}{}^{2}\rangle }{r}$$

(18) $$F_{\theta }=-\frac{\partial \langle \rho_{0}u_{1\theta }u_{1r}\rangle }{\partial r}-2\frac{1}{r}\frac{\partial \langle \rho_{0}u_{1\theta }{}^{2}\rangle }{\partial \theta }-\frac{\partial \langle \rho_{0}u_{1\theta }u_{1z}\rangle }{\partial z}-\frac{\langle \rho_{0}u_{1r}u_{1\theta }\rangle }{r}$$

(19) $$F_{z}=-\frac{\partial \langle \rho_{0}u_{1z}u_{1r}\rangle }{\partial r}-\frac{1}{r}\frac{\partial \langle \rho_{0}u_{1z}u_{1\theta }\rangle }{\partial \theta }-\frac{\partial \langle \rho_{0}u_{1z}{}^{2}\rangle }{\partial z}-\frac{\langle \rho_{0}u_{1r}u_{1z}\rangle }{r}$$

where *u_1r_*, *u_1θ_* and *u_1z_* are the *r*-, *θ*- and *z*-directional acoustic velocities in the 3D cylindrical coordinate system, respectively. Taking the 2D axisymmetric form of the driving force, the *r*- and *z*-directional components of ***F_R_*** can be simplified as

(20) $$F_{r}=-\frac{\partial \langle \rho_{0}u_{1r}{}^{2}\rangle }{\partial r}-\frac{\partial \langle \rho_{0}u_{1r}u_{1z}\rangle }{\partial z}-\frac{\langle \rho_{0}u_{1r}{}^{2}\rangle }{r}$$

(21) $$F_{z}=-\frac{\partial \langle \rho_{0}u_{1z}u_{1r}\rangle }{\partial r}-\frac{\partial \langle \rho_{0}u_{1z}{}^{2}\rangle }{\partial z}-\frac{\langle \rho_{0}u_{1r}u_{1z}\rangle }{r}$$

Compared with the expression of components of ***F_R_*** in the 2D rectangular coordinate system [84], nonlinear terms $-\langle \rho_{0}u_{1r}{}^{2}\rangle /r$ and $-\langle \rho_{0}u_{1r}u_{1z}\rangle /r$ are generated in *F_r_* and *F_z_*, respectively. Herein, to investigate the effect of these nonlinear terms on the acoustic streaming fields, we define the driving force of acoustic streaming without the nonlinear terms as ***F_R_*_1_**, and the components of ***F_R_*_1_** are expressed as

(22) $$F_{r1}=-\frac{\partial \langle \rho_{0}u_{1r}{}^{2}\rangle }{\partial r}-\frac{\partial \langle \rho_{0}u_{1r}u_{1z}\rangle }{\partial z}$$

(23) $$F_{z1}=-\frac{\partial \langle \rho_{0}u_{1z}u_{1r}\rangle }{\partial r}-\frac{\partial \langle \rho_{0}u_{1z}{}^{2}\rangle }{\partial z}$$

In this work, numerical simulations of acoustofluidic fields are carried out by the commercial FEM software COMSOL Multiphysics 5.3a (Burlington, MA, USA). The computational process consists of the following two steps. In the first step, acoustic field, which mainly includes the acoustic velocity field and acoustic pressure field, is computed according to the external actuation conditions and acoustic boundary conditions. In the second step, with the computed result of acoustic field, the acoustic streaming field is computed according to the fluidic boundary conditions. For the PM, the right-hand side of Equation (10) is included as a source term by adding a so-called weak contribution to the governing equations, and Equation (12) acts as a body force term. For the RSM, Equations (20) and (21) act as the body force terms in the *r* and *z* directions, respectively. More detailed descriptions of the FEM models will be shown in Section 3.

## 3. Numerical Models

### 3.1. The Ultrasonic Probe-Droplet-Substrate System for Micro/Nanoscale Particle Removal

**Figure 1:**
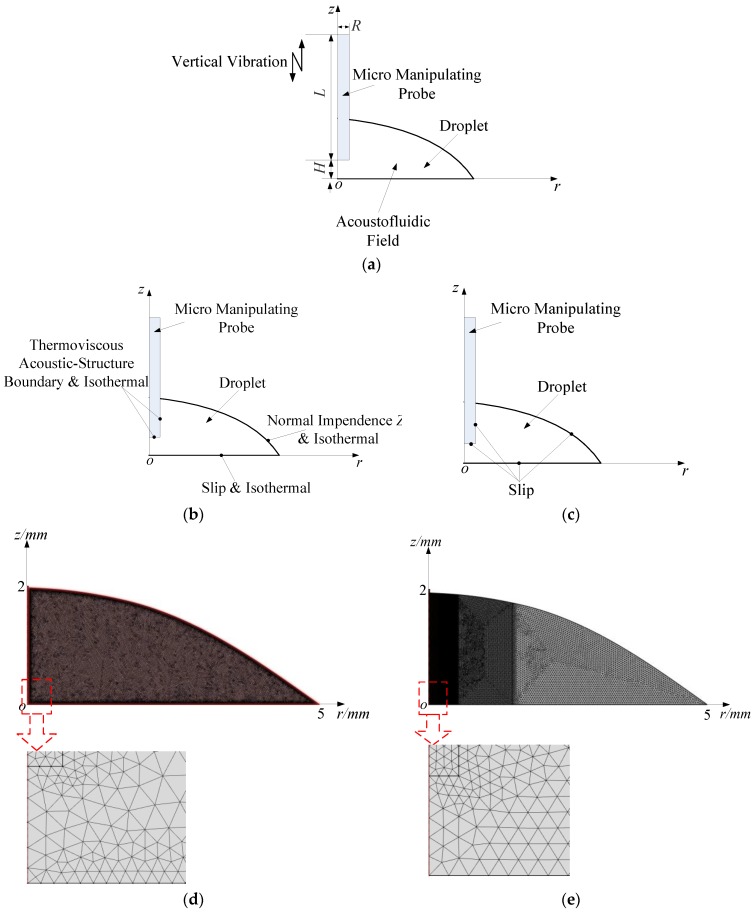
(**a**) 2D axisymmetric model of the acoustofluidic field in the ultrasonic probe-droplet-substrate system. (**b**) Boundary condition for the acoustic field in the ultrasonic probe-droplet-substrate system. (**c**) Boundary condition for the acoustic streaming field in the ultrasonic probe-droplet-substrate system. (**d**) Meshed model for the acoustofluidic field in the ultrasonic probe-droplet-substrate system under the simulation scheme of the PM. (**e**) Meshed model for the acoustofluidic field in the ultrasonic probe-droplet-substrate system under the simulation scheme of the RSM.

A numerical model for the ultrasonic probe-droplet-substrate system for micro/nanoscale particle removal is illustrated in Figure 1a. In COMSOL Multiphysics, the Thermoacoustics-Solid Mechanics coupled module is firstly used to compute the acoustic field by exciting the MMP in the *z* direction. The acoustic boundary condition is shown in Figure 1b, in which the normal impedance $Z_{0}=\rho_{air}c_{air}$ is adopted at the droplet-air interface; the droplet-substrate interface is set to be slip; the MMP-droplet interface is set to be the thermoviscous acoustic-structure boundary. All of the interfaces are set to be isothermal. In the Laminar Flow module for simulating the acoustic streaming field, the fluidic boundary condition is shown in Figure 1c, in which all of the interfaces are set to be slip [76,80,81,85,86,87]. 

The meshed FEM models of the ultrasonic probe-droplet-substrate system for the PM and RSM are shown in Figure 1d,e, respectively, in which the radius and height of the droplet are 5 mm and 2 mm, respectively. The maximum element size of the MMP is set to be 5 μm (1/3 of the radius of the MMP when *R* = 15 μm) in the models. 

For simulating the acoustofluidic field by the PM, as shown in Figure 1d, the computational mesh is generated from a maximum element size 2*δ* at the fluid domain boundaries and a maximum element size in the bulk of the fluid domain given by 10*δ*, where $\delta =\sqrt{\frac{2\mu }{\omega \rho_{0}}}$ is the viscous boundary layer thickness at 40 kHz (*ω* is the angular frequency of acoustic field) [1]. This mesh constitution scheme has proven to be effective and mesh converged and independent [61,62,72,88]. 

For simulating the acoustofluidic field by the RSM, since the driving force components (Equations (20) and (21)) contain nonlinear terms, the variation of the driving force for acoustic streaming may be drastic within the fluid domain near the MMP. Therefore, to precisely compute the driving force for acoustic streaming in the whole fluid domain as much as possible, based on the computation capability of the laptop (Lenovo E51-80-ISE, i7-6500U CPU, 16G RAM), the maximum element size of the fluid domain near the MMP is set to be 10 μm (about 0.34% of the wavelength of acoustic field at 40 kHz) in 0 < *r* < 0.5 mm region (Region 1), where removal of micro/nanoscale particles occurs. As shown in Figure 1e, the maximum element sizes of the rest fluid domain are set to be 20 μm (about 0.68% of the wavelength of acoustic field at 40 kHz) in 0.5 < *r* < 1.5 mm region (Region 2), and 40 μm (about 1.36% of the wavelength of acoustic field at 40 kHz) in 1.5 < *r* < 5 mm region (Region 3), respectively. The mesh independence analysis for this mesh constitution scheme can be found in the Supplementary Materials. 

### 3.2. The Droplet-Ultrasonic Substrate System for Micro/Nanoscale Particle Concentration

**Figure 2:**
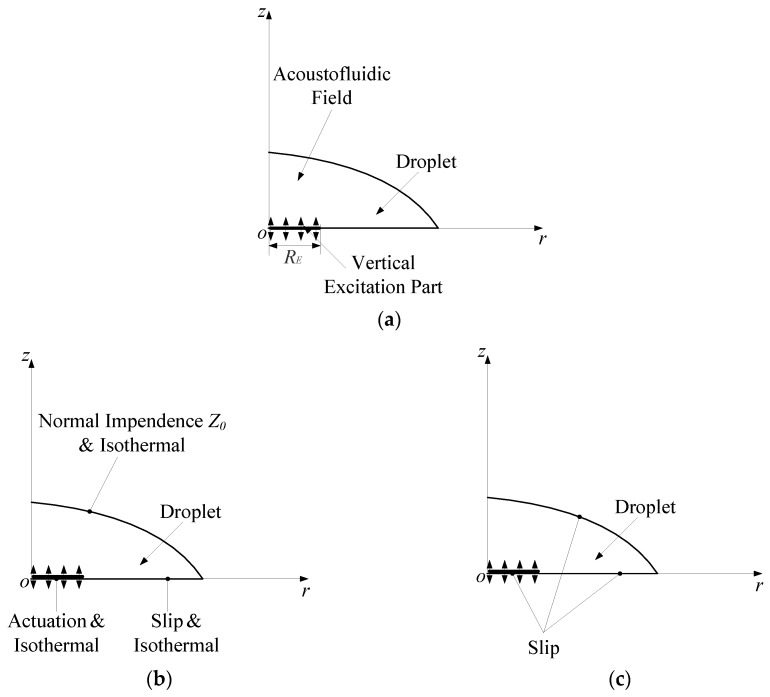

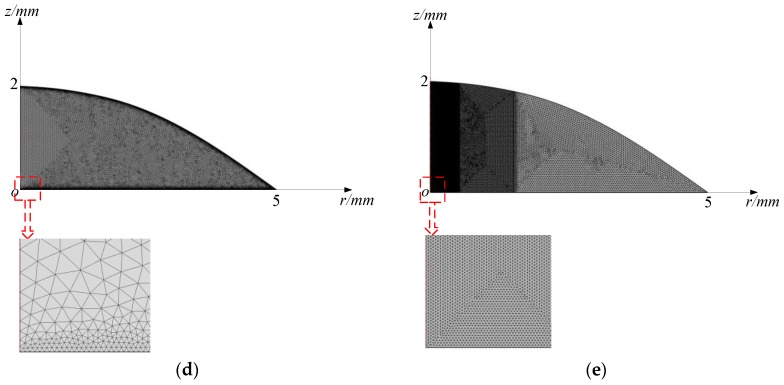
(**a**) 2D axisymmetric model of the acoustofluidic field in the droplet-ultrasonic substrate system. (**b**) Boundary condition for the acoustic field in the droplet-ultrasonic substrate system. (**c**) Boundary condition for the acoustic streaming field in the droplet-ultrasonic substrate system. (**d**) Meshed model for the acoustofluidic field in the droplet-ultrasonic substrate system under the simulation scheme of the PM. (**e**) Meshed model for the acoustofluidic field in the droplet-ultrasonic substrate system under the simulation scheme of the RSM.

A numerical model for the droplet-ultrasonic substrate system for micro/nanoscale particle concentration is illustrated in Figure 2a. The Thermoacoustics module is firstly utilized to compute the acoustic field by harmonically actuating the partial boundary of the fluid in the *z* direction. The acoustic and fluidic boundary conditions are shown in Figure 2b,c, respectively.

The meshed FEM models of the droplet-ultrasonic substrate system for the PM and RSM are shown in Figure 2d,e, respectively, in which the radius and height of the droplet are 5 mm and 2 mm, respectively.

For simulating the acoustofluidic field by the PM, as shown in Figure 2d, the computational mesh is generated from a maximum element size 2*δ* at the fluid domain boundaries and a maximum element size in the bulk of the fluid domain given by 10*δ*.

For simulating the acoustofluidic field by the RSM, as shown in Figure 2e, the maximum element size of the fluid domain is set to be 10 μm in 0 < *r* < 0.5 mm region (Region 1). In the rest fluid domain, the maximum element sizes are set to be 20 μm in 0.5 < *r* < 1.5 mm region (Region 2), and 40 μm in 1.5 < *r* < 5 mm region (Region 3), respectively. The mesh independence analysis for this mesh constitution scheme can also be found in the Supplementary Materials.

To discretize the computational models, linear, quadratic Lagrangian and quadratic Lagrangian shape functions are chosen for the acoustic pressure, acoustic velocity and temperature fields in the Thermoacoustics module, respectively. The quadratic serendipity elements are utilized to discretize the displacement field of solid in the Solid Mechanics module. In the Laminar Flow module, the discretization of Laminar Flow is set to be P3 + P2 elements (third-order elements for the velocity components and second-order elements for the pressure field). For the computation of acoustic field, the relative tolerance is set to be 0.001 in the Frequency Domain solver. For the computation of acoustic streaming field, the relative tolerance is also set to be 0.001 in the Steady State solver.

## 4. Experimental Verification

### 4.1. The Ultrasonic Probe-Droplet-Substrate System-Based Micro/Nanoscale Particle Removal

**Figure 3:**
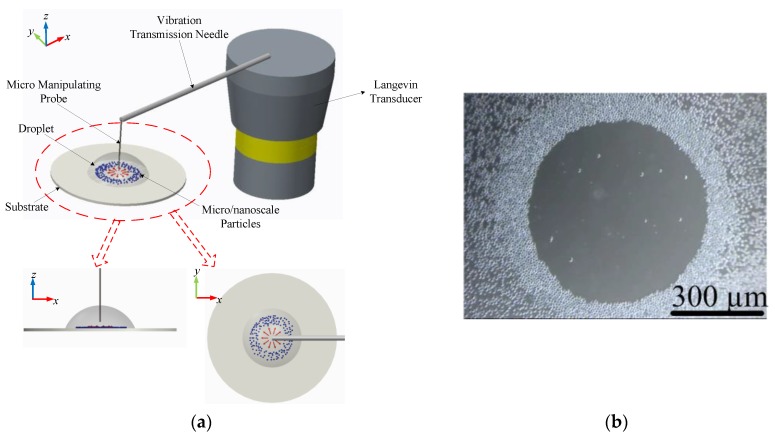

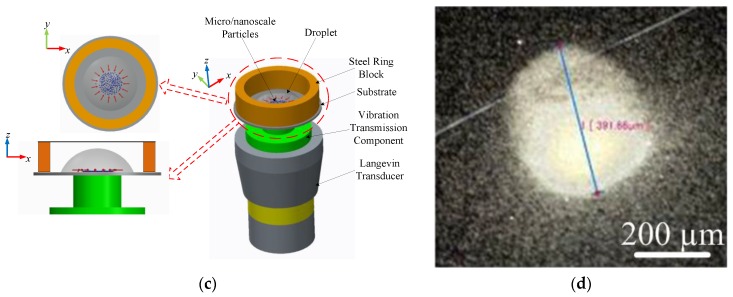
Experimental setups to verify the simulation results. (**a**) Schematic diagram of the ultrasonic probe-droplet-substrate system for micro/nanoscale particle removal. (**b**) Stable removal effect of yeast cells when *R* = 15 μm, *L* = 4 mm, *V_pvib_* = 0.3 m/s, *H* = 50 μm and *f_p_* = 40 kHz. (**c**) Schematic diagram of the droplet-ultrasonic substrate system for micro/nanoscale particle concentration. (**d**) Stable aggregation of AgNPs when *R_E_* = 3 mm, *V_svib_* = 10 mm/s and *f_s_* = 40 kHz.

In order to experimentally verify the FEM simulation results, an ultrasonic probe-droplet-substrate system for micro/nanoscale particle removal at the droplet-substrate interface is constructed. The experimental microscale particles (yeast cells, Angel Yeast Co., Ltd., Yichang, China) and nanoscale particles (Si nanoparticles, SiNPs, Beijing DK Nano Technology Co., Ltd., Beijing, China) have the average diameters of 4 μm and 400 nm, respectively. As shown in Figure 3a, the vibration transmission needle (VTN), which is made of nickel-plated steel (Shanghai Dongfeng Co., Ltd., Shanghai, China), is bonded to the radiation surface of a Langevin transducer (Suzhou Hainertec Co., Ltd., Suzhou, China), which vibrates with the piston mode [89]. Thus, the VTN can vibrate flexurally in the *z* direction. The MMP with the radius *R* and length *L*, which is made of glass fiber (Nanjing Fiberglass Research & Design Institute Co., Ltd., Nanjing, China), is bonded to the tip of the VTN, and thereby vibrates in the *z* direction with the vibration velocity amplitude *V_pvib_*. The MMP is inserted into the droplet of micro/nanoscale particle suspension on a silicon substrate (Zhejiang Lijing Co., Ltd., Hangzhou, China), and the distance between the MMP’s tip and substrate surface *H* is controlled by the manual X-Y-Z moving stage (LD125-LM-2, Shengling Precise Machinery Co., Ltd., Dongguan, China). The maximum height and radius of the droplet are 2 mm and 5 mm, respectively. The driving voltage for the device is sinusoidal, and the device works at its resonance frequency *f_p_*. Figure 3b shows the stable removal effect of yeast cells when *R* = 15 μm, *L* = 4 mm, *V_pvib_* = 0.3 m/s, *H* = 50 μm and *f_p_* = 40 kHz (the concentration of the yeast cell suspension is 1.38 mg/mL).

### 4.2. The Droplet-Ultrasonic Substrate System-Based Micro/Nanoscale Particle Concentration

A droplet-ultrasonic substrate system for micro/nanoscale particle concentration at the droplet-substrate interface is also constructed. The experimental microscale particles (yeast cells) and nanoscale particles (Ag nanoparticles, AgNPs, Beijing DK Nano Technology Co., Ltd., China) have the average diameters of 4 μm and 400 nm, respectively. As shown in Figure 3c, a vibration transmission component (VTC), whose radiation head’s radius is *R_E_*, is bonded to the radiation surface of a Langevin transducer, which vibrates with the piston mode in the *z* direction. The vibration velocity amplitude of the VTC’s radiation head is defined as *V_svib_*. A silicon substrate equipped with a steel ring block, which is used to restrain the flexural vibration of the substrate, is bonded to the VTC’s radiation head. A droplet of micro/nanoscale particle suspension, of which the maximum height and radius are 2 mm and 5 mm, respectively, is placed at the center of the substrate. The driving voltage for the device is sinusoidal, and the device works at its resonance frequency *f_s_*. Figure 3d shows the stable aggregation of AgNPs when *R_E_* = 3 mm, *V_svib_* = 10 mm/s and *f_s_* = 40 kHz (the concentration of the AgNP suspension is 2.78 mg/mL).

## 5. Results and Discussion

Unless otherwise specified, structural sizes, material parameters and working conditions of the manipulation systems listed in Table 1 are used in the simulations.

### 5.1. Simulated Acoustofluidic Fields in the Ultrasonic Probe-Droplet-Substrate System for Micro/Nanoscale Particle Removal

**Figure 4:**
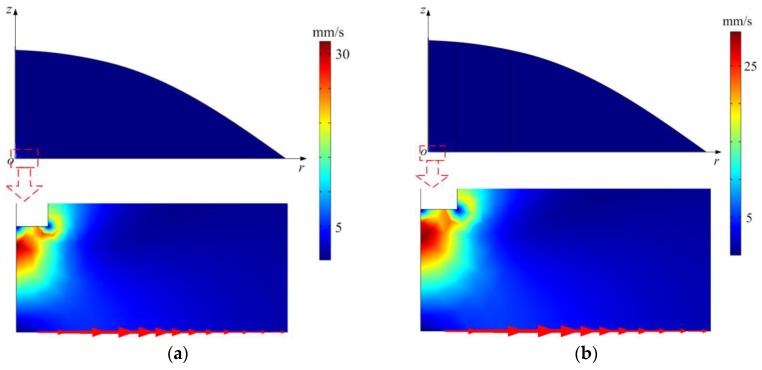

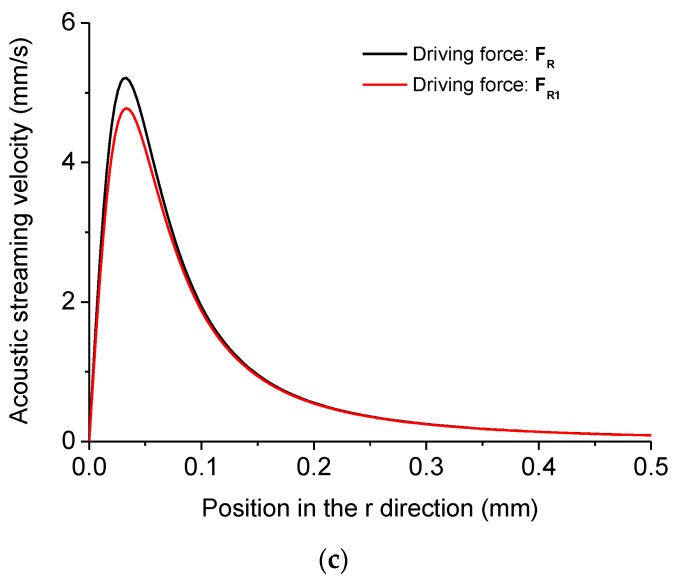
(**a**) Acoustic streaming field in the ultrasonic probe-droplet-substrate system computed by the PM. (**b**) Acoustic streaming field in the ultrasonic probe-droplet-substrate system computed by the RSM. (**c**) Computed acoustic streaming velocity distribution along the *r* direction at the droplet-substrate interface in the ultrasonic probe-droplet-substrate system when the driving forces of acoustic streaming are ***F_R_*** and ***F_R_*_1_**, respectively.

The simulated acoustic streaming fields in the ultrasonic probe-droplet-substrate system for micro/nanoscale particle removal by the PM and RSM are shown in Figure 4a,b, respectively, and the local enlarged images show the acoustic streaming patterns at the droplet-substrate interface near the MMP. It is seen from Figure 4a,b that as the acoustic streaming flows outwards from point *o*, micro/nanoscale particles can be dragged outwards and then a round cleaned area can be formed after a period of sonication (Figure 3b). Finally, a cleaned area with stable removal diameter (SRD) can be obtained, and the SRD is measured to be below millimeter scale.

In the ultrasonic probe-droplet-substrate system, we compare the drag force induced by acoustic streaming with the acoustic radiation force applied on the manipulated micro/nanoscale particles and find that acoustic streaming is dominant when the radius of manipulated particles falls between 100 nm and 10 microns (see Supplementary Materials). Thus, the effect of acoustic radiation force can be neglected in the ultrasonic probe-droplet-substrate system. The detailed information of the acoustic streaming fields will be discussed and analyzed in the following sections.

As emphasized in Section 2.2, to validate the significance and indispensability of the nonlinear terms in ***F_R_***, we compare the simulated acoustic streaming fields in the ultrasonic probe-droplet-substrate system when the driving forces of acoustic streaming are ***F_R_*** and ***F_R_*_1_**, respectively. Figure 4c shows the acoustic streaming velocity distribution at the droplet-substrate interface when the driving forces of acoustic streaming are ***F_R_*** and ***F_R_*_1_**, respectively. We extract the maximum acoustic streaming velocity *u_max_* in Figure 4c and the mean acoustic streaming velocity *u_mean_*, which is defined as

(24) $$u_{mean}=\frac{\int_{0}^{0.5\;\mathrm{mm}}u_{r}dr}{0.5\;\mathrm{mm}}$$

where *u_r_* is the acoustic streaming velocity at the *r* position. From Figure 4c, the deviations for *u_max_* and *u_mean_* are calculated to be 8.66% and 5.3%, respectively. Therefore, the nonlinear terms in ***F_R_*** are non-negligible for simulating acoustic streaming fields in the ultrasonic probe-droplet-substrate system by the RSM.

**Figure 5:**
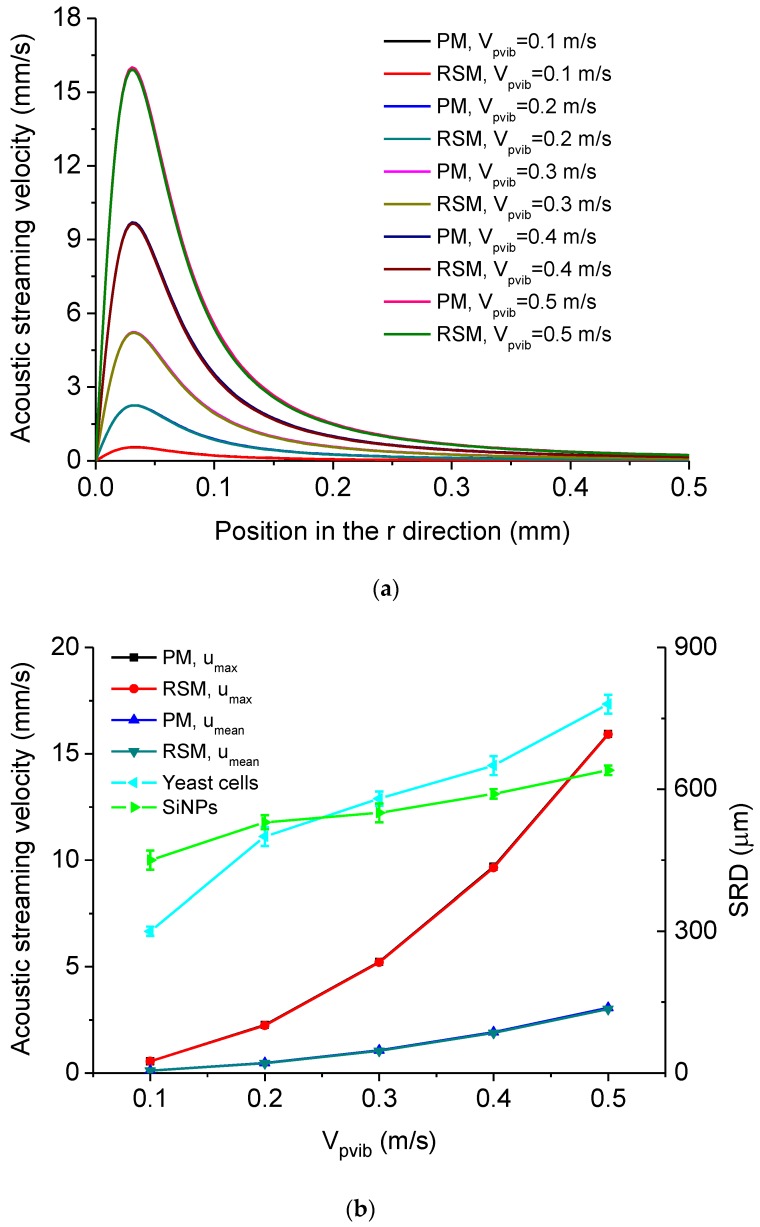

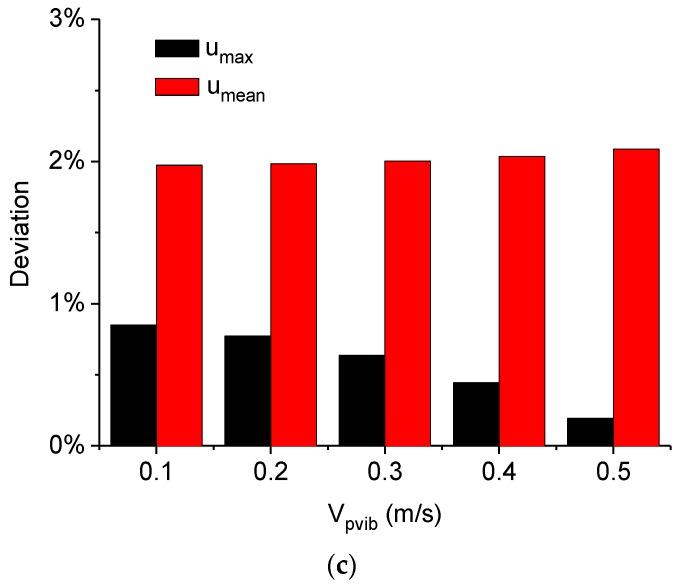
(**a**) Computed acoustic streaming velocity distribution along the *r* direction at the droplet-substrate interface in the ultrasonic probe-droplet-substrate system under different *V_pvib_*. (**b**) *u_max_*, *u_mean_* and SRD versus *V_pvib_* in the ultrasonic probe-droplet-substrate system. (**c**) Deviations of *u_max_* and *u_mean_* computed by the PM and RSM versus *V_pvib_* in the ultrasonic probe-droplet-substrate system.

The dependency of acoustic streaming velocity on the vibration velocity amplitude of the MMP *V_pvib_* is shown in Figure 5. Figure 5a shows the distribution of acoustic streaming velocity along the *r* direction at the droplet-substrate interface computed by the PM and RSM under different *V_pvib_*. It is seen that the acoustic streaming velocity firstly increases with the increase of *r* and then reaches *u_max_*, after which the acoustic streaming velocity decreases with the increase of *r* and tends to 0 when *r* = 0.5 mm. Figure 5b shows *u_max_*, *u_mean_* and experimentally measured SRD of the cleaned area for yeast cells and SiNPs versus *V_pvib_*. In Figure 5b, *V_pvib_* was controlled by changing the input power of the device (Figure 3a), and the standard deviation is obtained by five times of experimental measurements. It is seen that both *u_max_* and *u_mean_* increase with the increase of *V_pvib_*, which accounts for that the SRD increases with the increase of *V_pvib_*. In this case, one can realize the pinpoint or relatively large-area removal of micro/nanoscale particles [90] by easily tuning the input power of the device. Meanwhile, in Figure 5b, both *u_max_* and *u_mean_* computed by the PM are always larger than those computed by the RSM when *V_pvib_* is the same. To quantitatively demonstrate the deviation of acoustic streaming velocity computed by the PM and RSM, we define the deviation as

(25) $$D=\frac{u_{PM}-u_{RSM}}{u_{RSM}}\times 100\%$$

where *u_PM_* and *u_RSM_* are the simulated acoustic streaming velocities by the PM and RSM, respectively, and the results for *u_max_* and *u_mean_* versus *V_pvib_* are shown in Figure 5c. It is seen from Figure 5c that *D* of *u_max_* is always smaller than that of *u_mean_*. Also, *D* of *u_max_* decreases with the increase of *V_pvib_*, while *D* of *u_mean_* changes little. To physically understanding this result, we further process the driving force of acoustic streaming under the framework of the PM ***F_P_***. Firstly, we multiply both sides of Equation (6) by -***u*_1_** and take the time-averaged form. Then, we can obtain

(26) $$-\langle u_{1}\frac{\partial \rho_{1}}{\partial t}\rangle -\rho_{0}\langle u_{1}\nabla \cdot u_{1}\rangle =0$$

We add Equation (26) to Equation (12) and can obtain

(27) $$F_{P}=-\langle \rho_{1}\frac{\partial u_{1}}{\partial t}\rangle -\langle u_{1}\frac{\partial \rho_{1}}{\partial t}\rangle -\rho_{0}\langle u_{1}\nabla \cdot u_{1}\rangle -\rho_{0}\langle u_{1}\cdot \nabla u_{1}\rangle =-\langle \frac{\partial (\rho_{1}u_{1})}{\partial t}\rangle +F_{R}$$

Obviously, it is seen from Equation (27) that the difference of driving force of acoustic streaming between the PM and RSM is $-\langle \partial (\rho_{1}u_{1})/\partial t\rangle$, and we define it as ***F_add_***. The reason for that *D* of *u_max_* decreases with the increase of *V_pvib_* may be that as *V_pvib_* increases, i.e., the input power for the acoustic field increases, ***F_add_*** becomes less important compared to the main driving force of acoustic streaming (***F_R_***) in this system.

**Figure 6:**
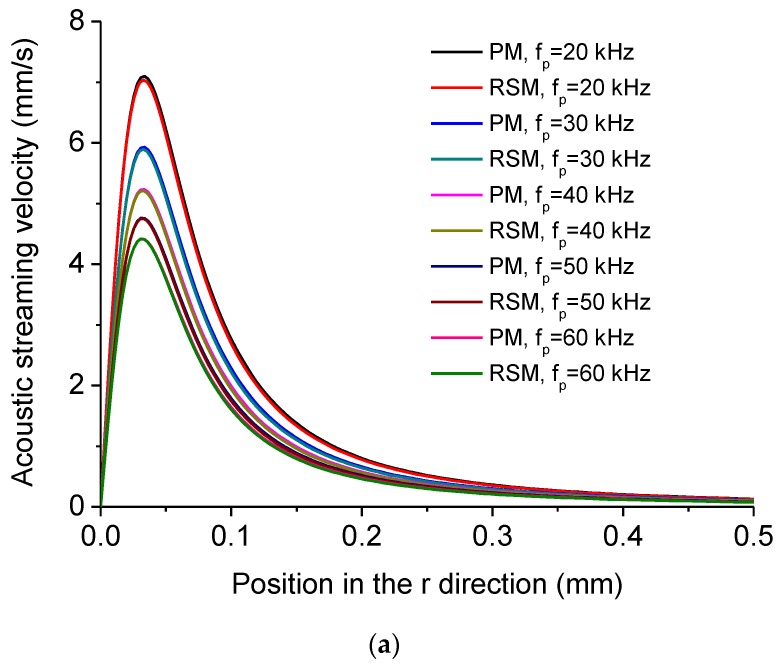

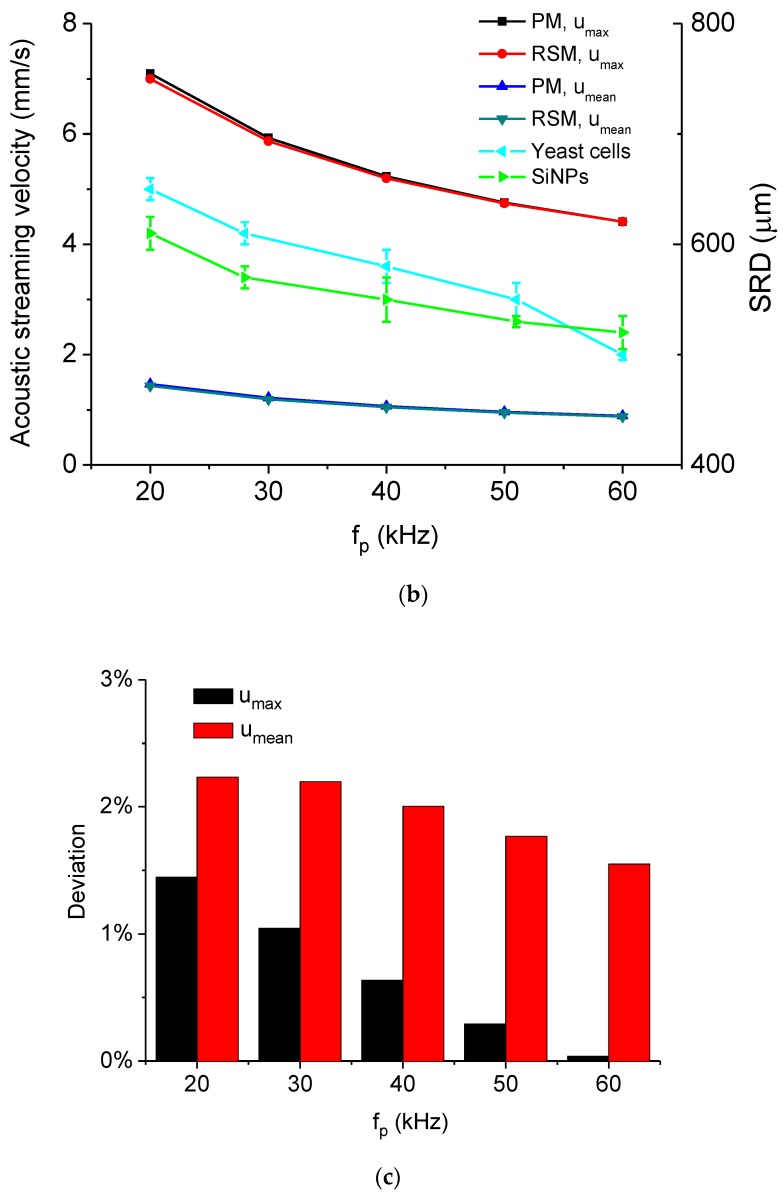
(**a**) Computed acoustic streaming velocity distribution along the *r* direction at the droplet-substrate interface in the ultrasonic probe-droplet-substrate system under different *f_p_*. (**b**) *u_max_*, *u_mean_* and SRD versus *f_p_* in the ultrasonic probe-droplet-substrate system. (**c**) Deviations of *u_max_* and *u_mean_* computed by the PM and RSM versus *f_p_* in the ultrasonic probe-droplet-substrate system.

The dependency of acoustic streaming velocity on the driving frequency of the system *f_p_* is shown in Figure 6. Figure 6a shows the distribution of acoustic streaming velocity along the *r* direction at the droplet-substrate interface computed by the PM and RSM under different *f_p_*. Figure 6b shows *u_max_*, *u_mean_* and experimentally measured SRD of the cleaned area for yeast cells and SiNPs versus *f_p_*. In Figure 6b, the SRD versus *f_p_* was measured with five Langevin transducers with different resonance frequencies (20 kHz, 28 kHz, 40 kHz, 51 kHz, 60 kHz). It is seen from Figure 6b that both *u_max_* and *u_mean_* decrease with the increase of *f_p_*, which accounts for that the SRD decreases with the increase of *f_p_*. This result indicates that the lower driving frequency is beneficial to removing micro/nanoscale particles within larger areas. Figure 6c shows *D* of *u_max_* and *D* of *u_mean_* versus *f_p_*, respectively, and it is seen that *D* of *u_max_* decreases with the increase of *f_p_*. This is because that although *V_pvib_* is kept the same, the higher input frequency corresponds to the higher input power for the acoustic field [91], in which case ***F_add_*** becomes less significant compared to ***F_R_*** when *f_p_* increases in this system.

**Figure 7:**
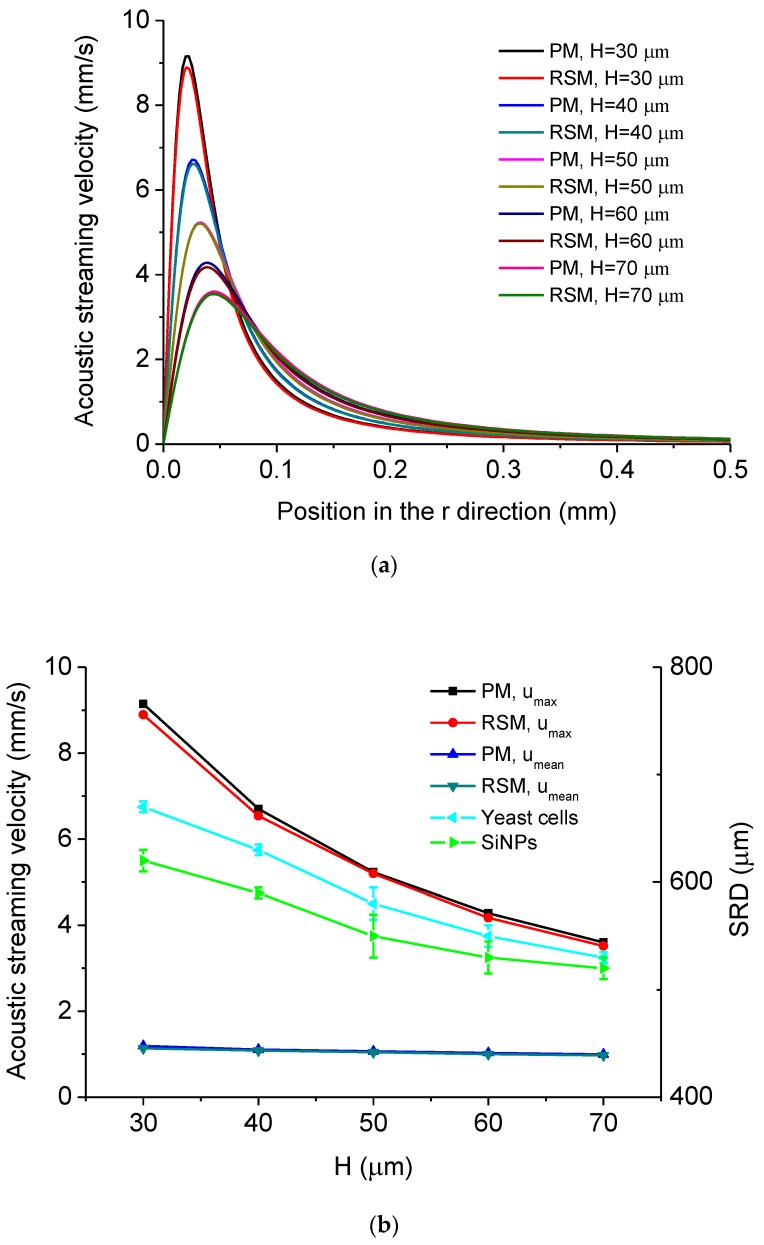

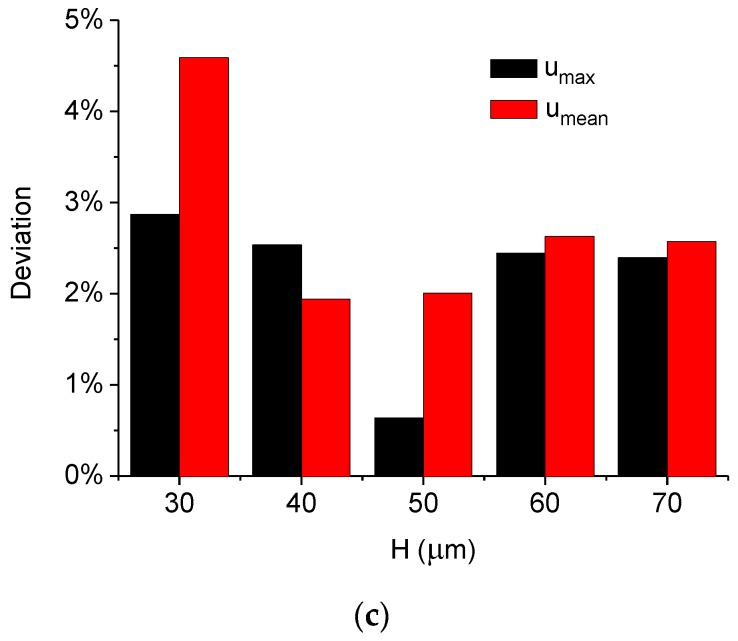
(**a**) Computed acoustic streaming velocity distribution along the *r* direction at the droplet-substrate interface in the ultrasonic probe-droplet-substrate system under different *H*. (**b**) *u_max_*, *u_mean_* and SRD versus *H* in the ultrasonic probe-droplet-substrate system. (**c**) Deviations of *u_max_* and *u_mean_* computed by the PM and RSM versus *H* in the ultrasonic probe-droplet-substrate system.

The dependency of acoustic streaming velocity on the distance between the MMP’s tip and substrate surface *H* is shown in Figure 7. Figure 7a shows the distribution of acoustic streaming velocity along the *r* direction at the droplet-substrate interface computed by the PM and RSM under different *H*, from which it is seen that the position where *u_max_* occurs moves outwards from point *o* when *H* increases. Figure 7b shows *u_max_*, *u_mean_* and experimentally measured SRD of the cleaned area for yeast cells and SiNPs versus *H*, from which it is seen that both *u_max_* and *u_mean_* decrease with the increase of *H*, which accounts for that the SRD decreases with the increase of *H*. In this case, one can realize the pinpoint or relatively large-area removal of micro/nanoscale particles just by tuning the distance between the MMP’s tip and substrate surface through controlling the X-Y-Z moving stage. Figure 7c shows *D* of *u_max_* and *D* of *u_mean_* versus *H*, respectively, and it is seen that *D* of *u_max_* is in the range of 0.6% to 2.9%, while *D* of *u_mean_* is in the range of 1.9% to 4.6%.

**Figure 8:**
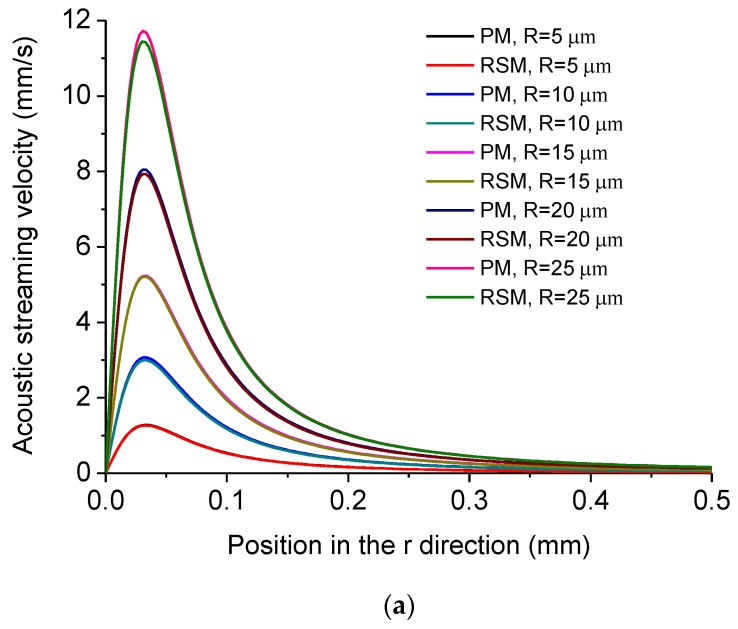

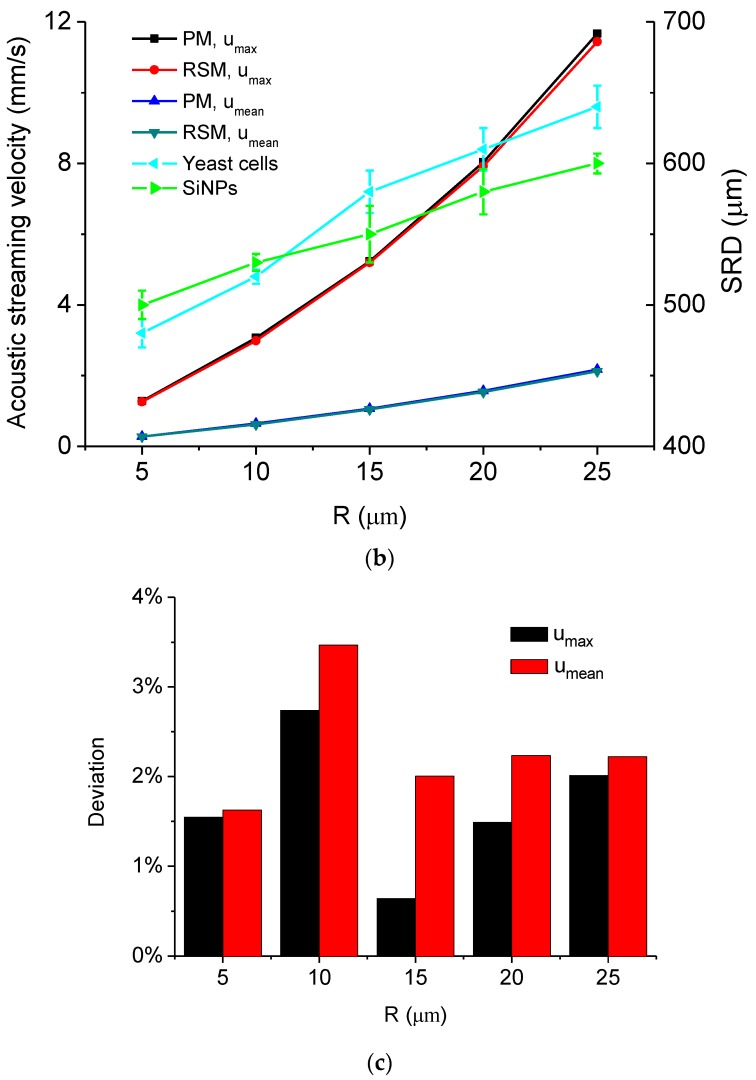
(**a**) Computed acoustic streaming velocity distribution along the *r* direction at the droplet-substrate interface in the ultrasonic probe-droplet-substrate system under different *R*. (**b**) *u_max_*, *u_mean_* and SRD versus *R* in the ultrasonic probe-droplet-substrate system. (**c**) Deviations of *u_max_* and *u_mean_* computed by the PM and RSM versus *R* in the ultrasonic probe-droplet-substrate system.

The dependency of acoustic streaming velocity on the radius of the MMP *R* is shown in Figure 8. Figure 8a shows the distribution of acoustic streaming velocity along the *r* direction at the droplet-substrate interface computed by the PM and RSM under different *R*. Figure 8b shows *u_max_*, *u_mean_* and experimentally measured SRD of the cleaned area for yeast cells and SiNPs versus *R*, from which it is seen that both *u_max_* and *u_mean_* increase with the increase of *R*, which results in that the SRD increases with the increase of *R*. This result indicates that the removal effect of micro/nanoscale particles can be controlled by using MMPs with different radii. Figure 8c shows *D* of *u_max_* and *D* of *u_mean_* versus *R*, respectively, and it is seen that *D* of *u_max_* is in the range of 0.6% to 2.8%, while *D* of *u_mean_* is in the range of 1.6% to 3.5%.

**Figure 9:**
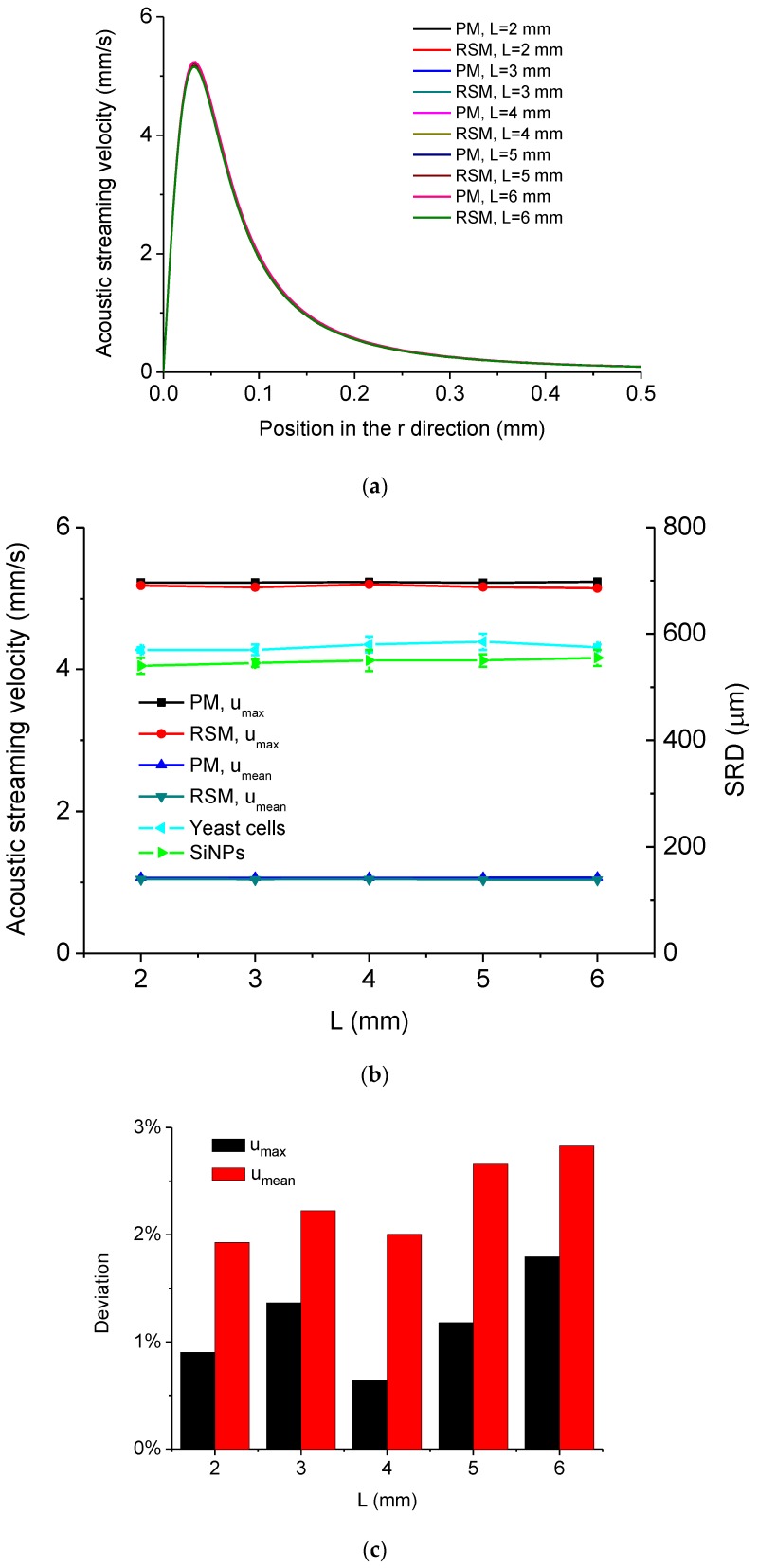
(**a**) Computed acoustic streaming velocity distribution along the *r* direction at the droplet-substrate interface in the ultrasonic probe-droplet-substrate system under different *L*. (**b**) *u_max_*, *u_mean_* and SRD versus *L* in the ultrasonic probe-droplet-substrate system. (**c**) Deviations of *u_max_* and *u_mean_* computed by the PM and RSM versus *L* in the ultrasonic probe-droplet-substrate system.

The dependency of acoustic streaming velocity on the length of the MMP *L* is shown in Figure 9. Figure 9a shows the distribution of acoustic streaming velocity along the *r* direction at the droplet-substrate interface computed by the PM and RSM under different *L*, from which it is seen that the distribution of acoustic streaming velocity changes little when *L* varies. Figure 9b shows *u_max_*, *u_mean_* and experimentally measured SRD of the cleaned area for yeast cells and SiNPs versus *L*, from which it is seen that *u_max_*, *u_mean_* and the SRD change little when *L* changes. This is because that the vibration of the MMP’s portion outside the droplet does not affect the acoustofluidic field within the droplet (Figure 1a and Figure 3a). As a result, it can be known that *L* has little influence on the removal effect of micro/nanoscale particles at the droplet-substrate interface in this system. Figure 9c shows *D* of *u_max_* and *D* of *u_mean_* versus *L*, respectively, and it is seen that *D* of *u_max_* is in the range of 0.6% to 1.8%, while *D* of *u_mean_* is in the range of 1.9% to 2.9%.

### 5.2. Simulated Acoustofluidic Fields in the Droplet-Ultrasonic Substrate System for Micro/Nanoscale Particle Concentration

**Figure 10:**
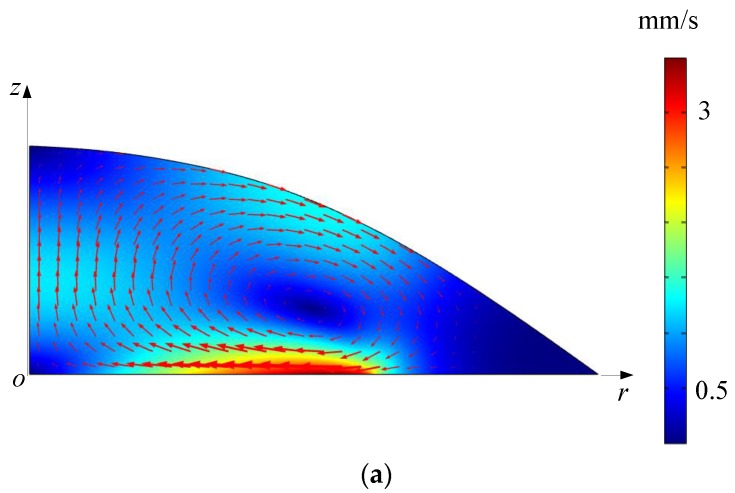

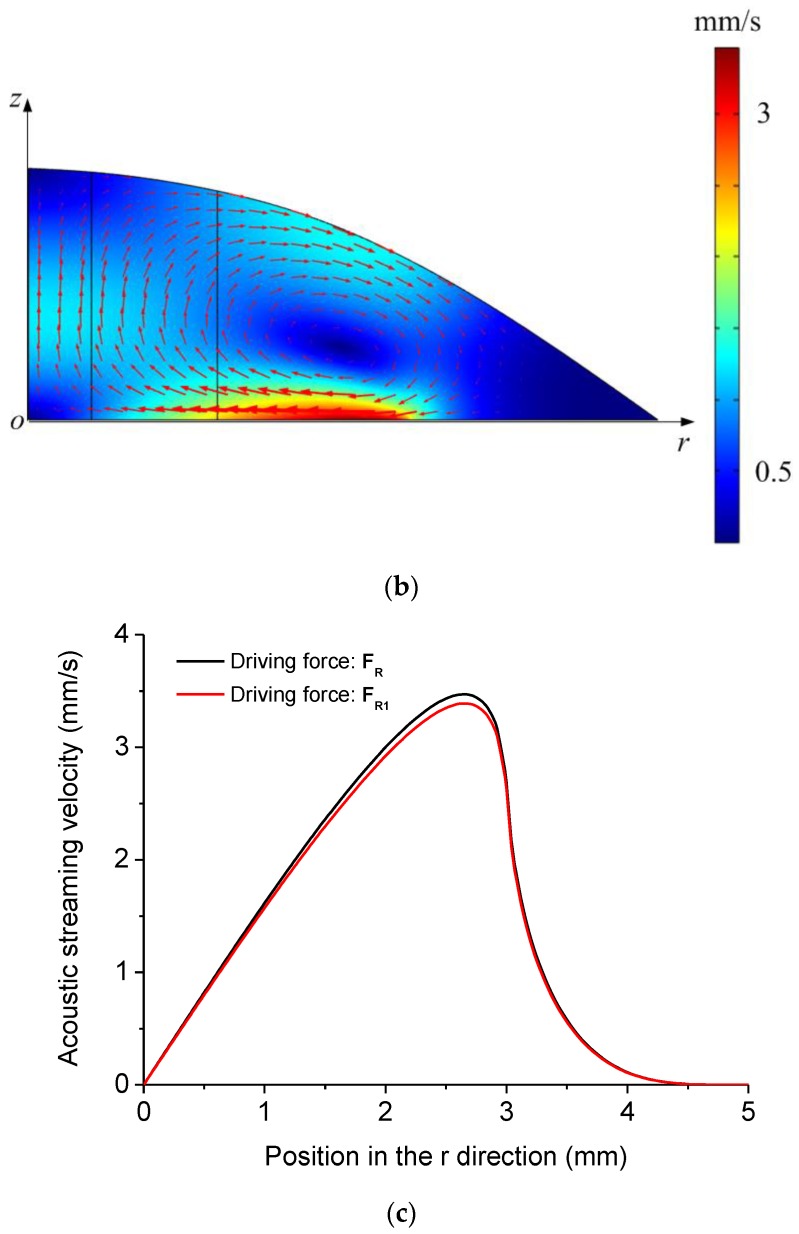
(**a**) Acoustic streaming field in the droplet-ultrasonic substrate system computed by the PM. (**b**) Acoustic streaming field in the droplet-ultrasonic substrate system computed by the RSM. (**c**) Computed acoustic streaming velocity magnitude distribution along the *r* direction at the droplet-substrate interface in the droplet-ultrasonic substrate system when the driving forces of acoustic streaming are ***F_R_*** and ***F_R_*_1_**, respectively.

The simulated acoustic streaming fields in the droplet-ultrasonic substrate system for micro/nanoscale particle concentration by the PM and RSM are shown in Figure 10a,b, respectively, from which it is seen that there exist vortices in the droplet. At the droplet-substrate interface, as the acoustic streaming flows from the outside boundary of the droplet to point *o*, micro/nanoscale particles can be dragged to point *o*, and be concentrated in the central area (Figure 3c). Finally, the stable concentration diameter (SCD) of aggregation of micro/nanoscale particles can be reached.

In the droplet-ultrasonic substrate system, we also compare the drag force induced by acoustic streaming with the acoustic radiation force applied on the manipulated micro/nanoscale particles and find that acoustic streaming is also dominant when the radius of manipulated particles falls between 100 nm and 10 microns (see Supplementary Materials). Thus, the effect of acoustic radiation force can also be neglected in the droplet-ultrasonic substrate system. The detailed information of the acoustic streaming fields will be discussed and analyzed in the following sections.

We also compare the simulated acoustic streaming fields in the droplet-ultrasonic substrate system when the driving forces of acoustic streaming are ***F_R_*** and ***F_R_*_1_**, respectively. Figure 10 shows the acoustic streaming velocity magnitude distribution at the droplet-substrate interface when the driving forces of acoustic streaming are ***F_R_*** and ***F_R_*_1_**, respectively. We extract the maximum acoustic streaming velocity magnitude *u_MAX_* in Figure 10c and the mean acoustic streaming velocity magnitude *u_MEAN_*, which is defined as

(28) $$u_{MEAN}=\frac{\int_{0}^{5\;\mathrm{mm}}u_{R}dr}{5\;\mathrm{mm}}$$

where *u_R_* is the acoustic streaming velocity magnitude at the *r* position. From Figure 10c, the deviations for *u_MAX_* and *u_MEAN_* are calculated to be 2.32% and 2.59%, respectively. Therefore, the nonlinear terms in ***F_R_*** are also non-negligible for simulating acoustic streaming fields in the droplet-ultrasonic substrate system by the RSM.

**Figure 11:**
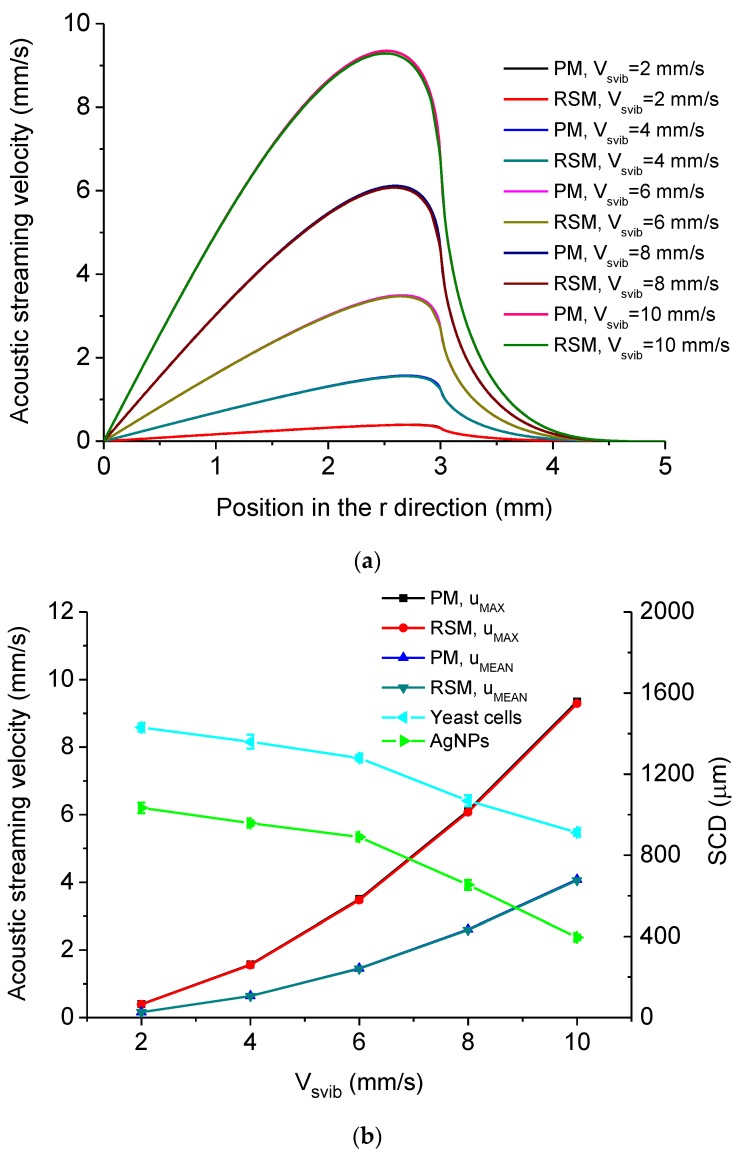

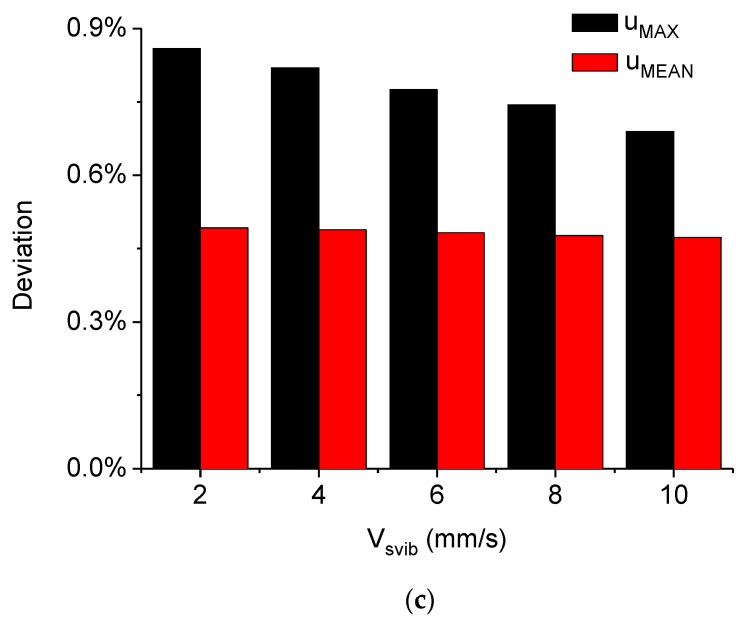
(**a**) Computed acoustic streaming velocity magnitude distribution along the *r* direction at the droplet-substrate interface in the droplet-ultrasonic substrate system under different *V_svib_*. (**b**) *u_MAX_*, *u_MEAN_* and SCD versus *V_svib_* in the droplet-ultrasonic substrate system. (**c**) Deviations of *u_MAX_* and *u_MEAN_* computed by the PM and RSM versus *V_svib_* in the droplet-ultrasonic substrate system.

The dependency of acoustic streaming velocity magnitude on the excitation velocity amplitude *V_svib_* is shown in Figure 11. Figure 11a shows the distribution of acoustic streaming velocity magnitude along the *r* direction at the droplet-substrate interface computed by the PM and RSM under different *V_svib_*. It is seen that the acoustic streaming velocity magnitude firstly increases with the increase of *r*, and then reaches *u_MAX_*, after which the acoustic streaming velocity magnitude decreases with the increase of *r* and tends to 0 when *r* = 5 mm. Also, the position where *u_MAX_* occurs moves inwards when *V_svib_* increases. Figure 11b shows *u_MAX_*, *u_MEAN_* and experimentally measured SCD of aggregation of yeast cells and AgNPs versus *V_svib_*. In Figure 11b, *V_svib_* was controlled by changing the input power of the device (Figure 3c), and the standard deviation is obtained by five times of experimental measurements. It is seen in Figure 11b that both *u_MAX_* and *u_MEAN_* increase with the increase of *V_svib_*, which accounts for that the SCD decreases with the increase of *V_svib_*. In this case, by easily tuning the input power of the device, one can tune the scale and density of aggregation of micro/nanoscale particles, both of which have significant applications in biochemical sensing [32] and assembly/fabrication of micro/nano devices [9]. Figure 11c shows *D* of *u_MAX_* and *D* of *u_MEAN_* computed by the PM and RSM versus *V_svib_*, respectively, from which it is seen that *D* of *u_MAX_* is always larger than that of *u_MEAN_*. It is also seen from Figure 11c that *D* of *u_MAX_* decreases with the increase of *V_svib_*. This is because that as *V_svib_* increases, i.e., the input power for the acoustic field increases, ***F_add_*** becomes less important compared to the main driving force of acoustic streaming (***F_R_***) in this system.

**Figure 12:**
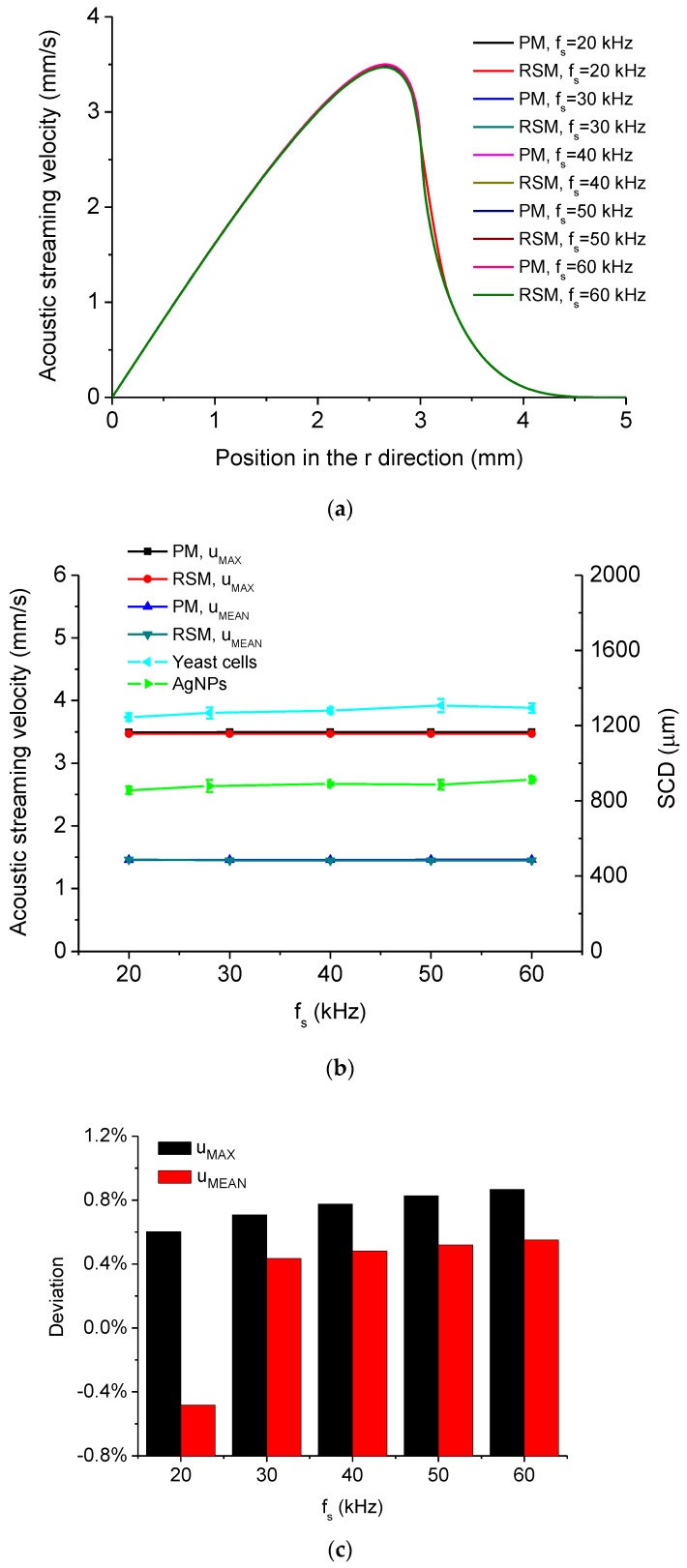
(**a**) Computed acoustic streaming velocity magnitude distribution along the *r* direction at the droplet-substrate interface in the droplet-ultrasonic substrate system under different *f_s_*. (**b**) *u_MAX_*, *u_MEAN_* and SCD versus *f_s_* in the droplet-ultrasonic substrate system. (**c**) Deviations of *u_MAX_* and *u_MEAN_* computed by the PM and RSM versus *f_s_* in the droplet-ultrasonic substrate system.

The dependency of acoustic streaming velocity magnitude on the driving frequency of the system *f_s_* is shown in Figure 12. Figure 12a shows the distribution of acoustic streaming velocity magnitude along the *r* direction at the droplet-substrate interface computed by the PM and RSM under different *f_s_*, from which it is seen that the distributions of acoustic streaming velocity magnitudes change little when *f_s_* varies. Figure 12b shows *u_MAX_*, *u_MEAN_* and experimentally measured SCD of aggregation of yeast cells and AgNPs versus *f_s_*. In Figure 12b, the SCD versus *f_s_* was measured with five Langevin transducers with different resonance frequencies (20 kHz, 28 kHz, 40 kHz, 51 kHz, 60 kHz). It is seen from Figure 12b both *u_MAX_* and *u_MEAN_* change little when *f_s_* varies, which accounts for that the SCD changes little when *f_s_* varies. Therefore, it can be known that the driving frequency of the device has little influence on the concentration effect of micro/nanoscale particles at the droplet-substrate interface in this system. Figure 12c shows *D* of *u_MAX_* and *D* of *u_MEAN_* versus *f_s_*, respectively, from which it is seen that *D* of *u_MAX_* increases with the increase of *f_s_*. This may result from that when *f_s_* increases, ***F_add_*** (generated from the time average of the partial derivative of *−ρ*_1_***u***_1_ with respect to time) becomes more important compared to the main driving force of acoustic streaming (***F_R_***) in this system.

**Figure 13:**
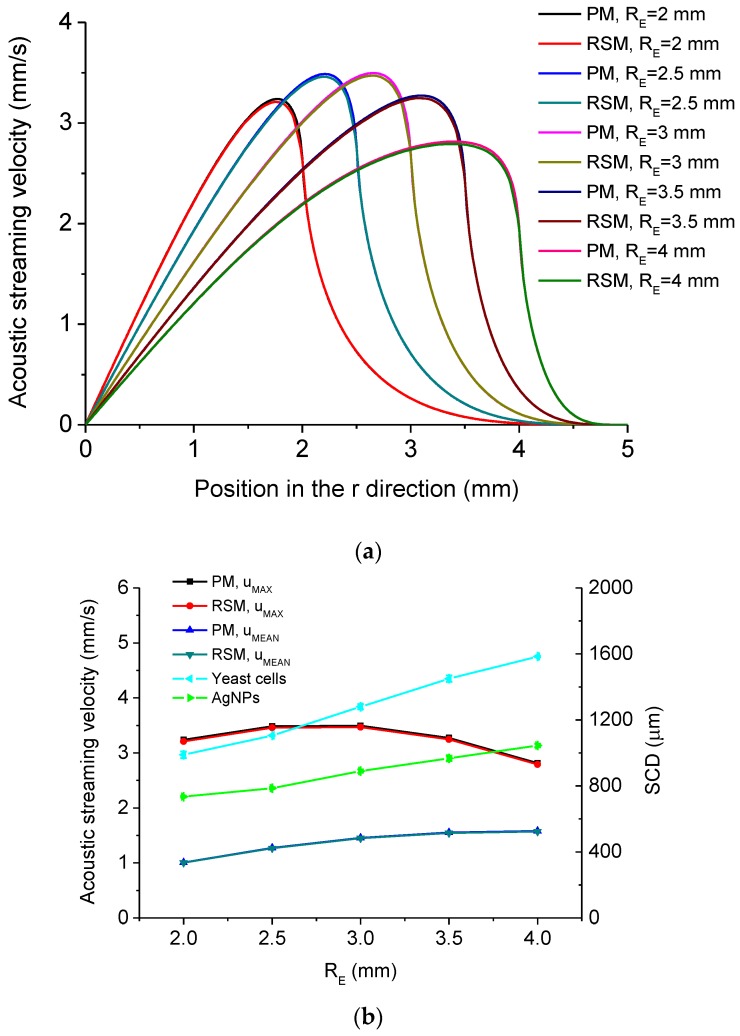

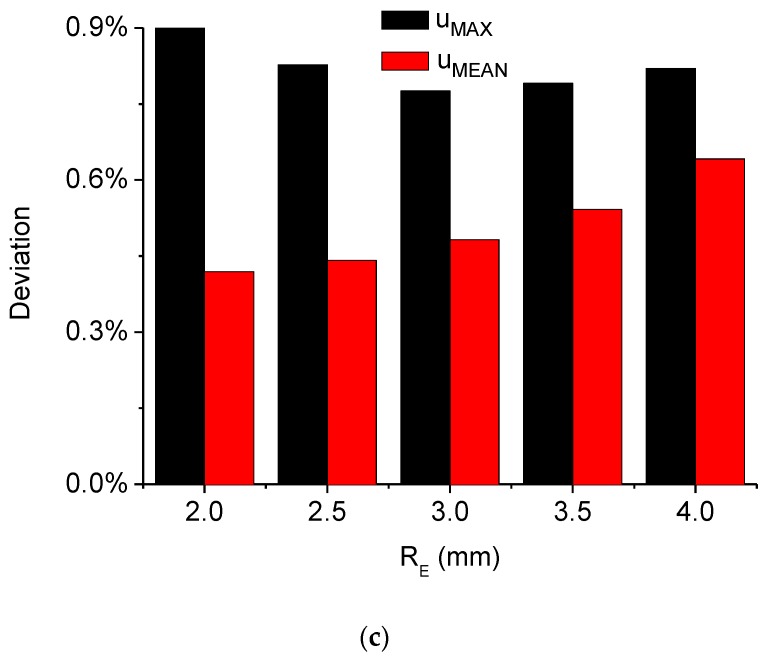
(**a**) Computed acoustic streaming velocity magnitude distribution along the *r* direction at the droplet-substrate interface in the droplet-ultrasonic substrate system under different *R_E_*. (**b**) *u_MAX_*, *u_MEAN_* and SCD versus *R_E_* in the droplet-ultrasonic substrate system. (**c**) Deviations of *u_MAX_* and *u_MEAN_* computed by the PM and RSM versus *R_E_* in the droplet-ultrasonic substrate system.

The dependency of acoustic streaming velocity magnitude on the radius of excitation part *R_E_* is shown in Figure 13. Figure 13a shows the distribution of acoustic streaming velocity magnitude along the *r* direction at the droplet-substrate interface computed by the PM and RSM under different *R_E_*, from which it is seen that the position where *u_MAX_* occurs moves outwards from point *o* when *R_E_* increases. Figure 13b shows *u_MAX_*, *u_MEAN_* and experimentally measured SCD of aggregation of yeast cells and AgNPs versus *R_E_*. In Figure 13b, the SCD versus *R_E_* was measured with VTCs with different radiation heads’ radii (Figure 3c). It is seen from Figure 13b that *u_MAX_* firstly increases with the increase of *R_E_* and then decreases with the increase of *R_E_*, while *u_MEAN_* increases with the increase of *R_E_*. It is also seen from Figure 13b that the SCD increases with the increase of *R_E_*. This may result from that when *R_E_* increases, the coverage area of the inward acoustic streaming flow increases (Figure 13a), which is beneficial to concentrating micro/nanoscale particles within larger areas. Figure 13c shows *D* of *u_MAX_* and *D* of *u_MEAN_* versus *R_E_*, respectively, and it is seen that the *D* of *u_MAX_* is in the range of 0.77% to 0.92%, while *D* of *u_MEAN_* is in the range of 0.41% to 0.65%.

## 6. Conclusions

We have numerically simulated and analyzed the 2D axisymmetric acoustofluidic fields in the ultrasonic probe-droplet-substrate system and droplet-ultrasonic substrate system by the PM and RSM, respectively. It is found that in the ultrasonic probe-droplet-substrate system for micro/nanoscale particle removal, the acoustic streaming field changes with the variation of vibration velocity of the MMP, driving frequency of the device, distance between the MMP’s tip and substrate surface, and radius of the MMP. It is also found that in the droplet-ultrasonic substrate system for micro/nanoscale particle concentration, the acoustic streaming field changes with the variation of excitation velocity and length of excitation part. It is also shown that there exist small differences between the simulation results of acoustic streaming fields computed by the PM and RSM. The demonstrated simulation results in this paper may provide some useful guidelines for controlling acoustic streaming fields in the droplet-based acoustofluidic systems.

## Figures and Tables

**Table 1:** Structural sizes, material parameters and working conditions of the manipulation systems used in the simulations.

Height of the Droplet (mm)	Radius of the Droplet (mm)	Density of Water *ρ*_0_ (kg/m^3^)
2	5	998
Sound speed in water *c_0_* (m/s)	Shear viscosity of water *μ* (Pa∙s)	Bulk-to-shear viscosity ratio in water *μ_b_*/*μ*
1482	0.001	2.1
Driving frequency of the ultrasonic probe-droplet-substrate system *f_p_* (kHz)	Vibration velocity of the MMP *V_pvib_* (m/s)	Distance between the MMP’s tip and substrate surface *H* (μm)
40	0.3	50
Radius of the MMP *R* (μm)	Length of the MMP *L* (mm)	Density of the MMP (kg/m^3^)
15	4	2210
Young’s modulus of the MMP (Pa)	Poisson ratio of the MMP	Driving frequency of the droplet-ultrasonic substrate system *f_s_* (kHz)
7.2 × 10^10^	0.2	40
Excitation velocity of the droplet-ultrasonic substrate system *V_svib_* (mm/s)	Radius of the excitation part *R_E_* (mm)	Density of yeast cells (kg/m^3^)
6	3	1114
Sound speed in yeast cells (m/s)	Density of SiNPs (kg/m^3^)	Sound speed in SiNPs (m/s)
1606	2330	5664
Density of AgNPs (kg/m^3^)	Sound speed in AgNPs (m/s)	Sound speed in air *c_air_* (m/s)
10,500	2600	343
Density of air *ρ_air_* (kg/m^3^)		
1.205		

## Supplementary Materials

The following are available online at https://www.mdpi.com/2072-666X/11/1/22/s1, Figure S1 (a) Acoustic streaming velocity distribution along the *r* direction at the droplet-substrate interface in Region 1 in the ultrasonic probe-droplet-substrate system under different mesh constitutions computed by the RSM. (b) *u_max_* and *u_mean_* at the droplet-substrate interface in Region 1 in the ultrasonic probe-droplet-substrate system under different mesh constitutions computed by the RSM. Figure S2 (a) Acoustic streaming velocity magnitude distribution along the *r* direction at the droplet-substrate interface in the droplet-ultrasonic substrate system under different mesh constitutions computed by the RSM. (b) *u_MAX_* and *u_MEAN_* at the droplet-substrate interface in the droplet-ultrasonic substrate system under different mesh constitutions computed by the RSM. Figure S3 (a) Computed acoustic radiation force field for yeast cells in the ultrasonic probe-droplet-substrate system under the mesh constitution for the PM. (b) Computed acoustic radiation force field for SiNPs in the ultrasonic probe-droplet-substrate system under the mesh constitution for the PM. Figure S4 (a) Computed acoustic radiation force field for yeast cells in the droplet-ultrasonic substrate system under the mesh constitution for the PM. (b) Computed acoustic radiation force field for AgNPs in the droplet-ultrasonic substrate system under the mesh constitution for the PM.
