# Supplementary material for: Modeling and Analysis of the Two-Dimensional Axisymmetric Acoustofluidic Fields in the Probe-Type and Substrate-Type Ultrasonic Micro/Nano Manipulation Systems
Source: Micromachines (Basel). 2019 Dec 24;11(1):22. doi: 10.3390/mi11010022 (PMC7019555; doi:10.3390/mi11010022)
Supplement: Supplementary file 1 [file micromachines-11-00022-s001.pdf]

micromachines

MDPI

Supplementary Material

Modeling and Analysis of the Two-Dimensional Axisymmetric Acoustofluidic Fields in the Probe-Type and Substrate-Type Ultrasonic Micro/Nano Manipulation Systems

Pengzhan Liu [1,2,*], Qiang Tang [3], Songfei Su [4], Jie Hu [5] and Yang Yu [6,*]

1 State Key Lab of Mechanics and Control of Mechanical Structures, Nanjing University of Aeronautics and Astronautics, Nanjing 210016, China
2 Department of Mechanical Engineering and Materials Science, Duke University, Durham, NC 27708, USA
3 Faculty of Mechanical and Material Engineering, Huaiyin Institute of Technology, Huaian 223003, China
4 School of Mechanical Engineering, Nanjing Institute of Technology, Nanjing 211167, China
5 School of Engineering, Jiangxi Agricultural University, Nanchang 330045, China
6 School of Civil and Environmental Engineering, University of Technology Sydney, Sydney, NSW 2007, Australia
* Correspondence: pengzhanliu@nuaa.edu.cn (P.L.); yang.yu@uts.edu.au (Y.Y.)

1. Mesh Dependence Analysis for the Acoustic Streaming Field in the Ultrasonic Probe-Droplet-Substrate System Computed by the RSM

The mesh sensitive study is performed to make sure the numerical results of acoustic streaming fields in the ultrasonic probe-droplet-substrate system are independent of the mesh size. Given the computation capability of our laptop (only 16G RAM), we keep the ratio of maximum element sizes in Region 1, Region 2 and Region 3 as 1: 2: 4, and choose the maximum element sizes in Region 1 from 7 μm to 11 μm. The computed acoustic streaming velocity distribution at the droplet-substrate interface in Region 1 is presented in Figure S1a, and u_{max} and u_{mean} versus maximum element size in Region 1 are shown in Figure S1b. From Figure S1b, the maximum deviations for u_{max} and u_{mean} under different mesh constitutions computed by the RSM are calculated to be 0.9% and 0.47%, respectively. In this case, it is reasonable to choose the mesh constitution scheme for the ultrasonic probe-droplet-substrate system for the RSM, which has been described in Section 3.1.

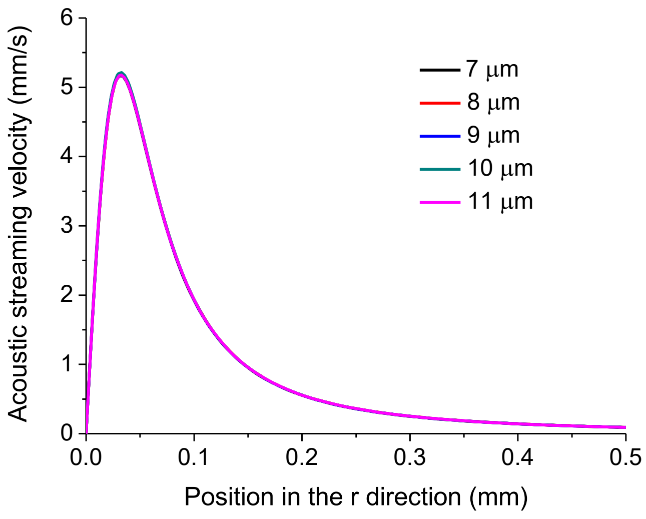

(a)

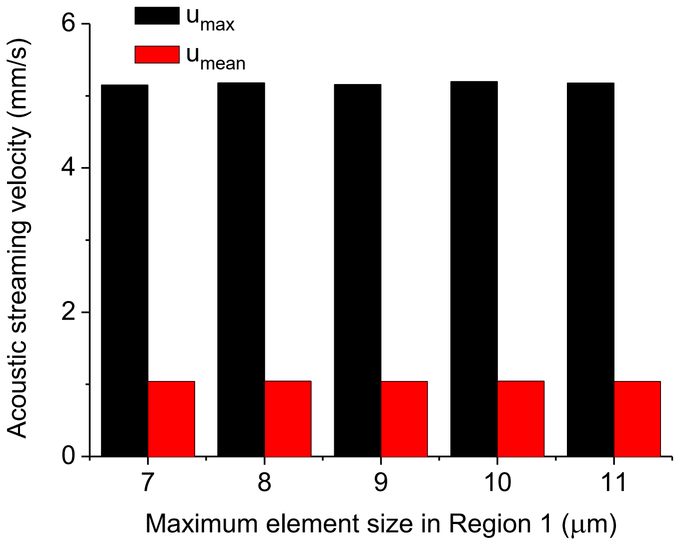

(**b**)

Figure S1. (**a**) Acoustic streaming velocity distribution along the *r* direction at the droplet-substrate interface in Region 1 in the ultrasonic probe-droplet-substrate system under different mesh constitutions computed by the RSM. (**b**) u_{max} and u_{mean} at the droplet-substrate interface in Region 1 in the ultrasonic probe-droplet-substrate system under different mesh constitutions computed by the RSM.

2. Mesh Dependence Analysis for the Acoustic Streaming Field in the Droplet-Ultrasonic Substrate System Computed by the RSM

We also perform the mesh independence analysis for the simulation results of acoustic streaming fields in the droplet-ultrasonic substrate system computed by the RSM. We also keep the ratio of maximum element sizes in Region 1, Region 2 and Region 3 as 1: 2: 4, and choose the maximum element sizes in Region 1 from 7 μm to 11 μm. The computed acoustic streaming velocity magnitude distribution at the droplet-substrate interface is presented in Figure S2a, and u_{MAX} and u_{MEAN} versus maximum element size are shown in Figure S2b. From Figure S2b, the maximum deviations for u_{MAX} and u_{MEAN} under different mesh constitutions computed by the RSM are calculated to be 0.46% and 0.3%, respectively. In this case, it is also reasonable to choose the mesh constitution scheme for the droplet-ultrasonic substrate system for the RSM, which has been described in Section 3.2.

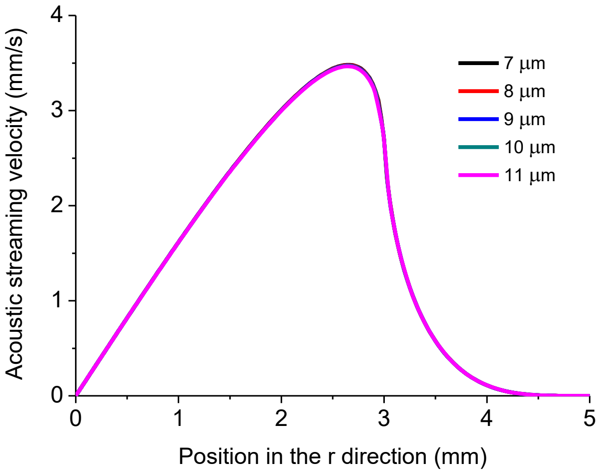

(**a**)

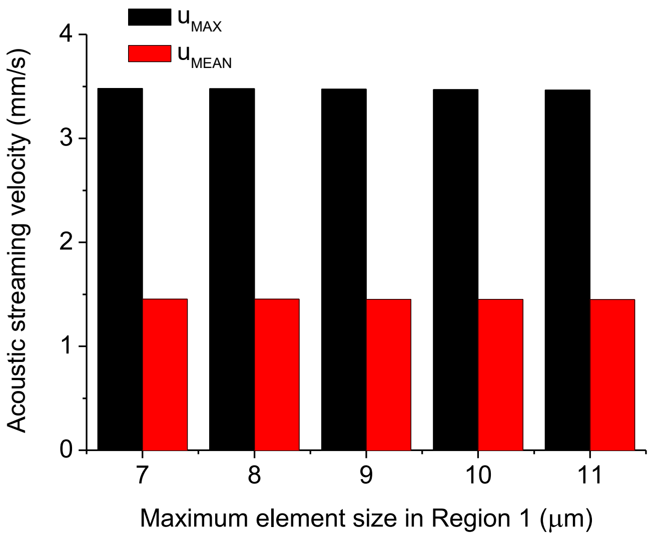

(**b**)

Figure S2. (**a**) Acoustic streaming velocity magnitude distribution along the *r* direction at the droplet-substrate interface in the droplet-ultrasonic substrate system under different mesh constitutions computed by the RSM. (**b**) u_{MAX} and u_{MEAN} at the droplet-substrate interface in the droplet-ultrasonic substrate system under different mesh constitutions computed by the RSM.

3. Comparison between Acoustic Streaming Induced Drag Force and Acoustic Radiation Force in the Ultrasonic Probe-Droplet-Substrate System

Herein, we calculate the drag force along the *r* axis caused by the *r*-directional acoustic streaming flow and the acoustic radiation force at the droplet-substrate interface, respectively. The calculation formula of drag force [1] is

$$F_{rdrag} = 6\pi\mu R_s (u_r - u_p) \quad \text{(S1)}$$

where R_s is the average radius of the manipulated particles, and u_r and u_p are the acoustic streaming velocity magnitude and particle velocity at the *r* position, respectively. In Equation (S1), the particle velocity u_p is set to be zero for only consideration of the initial circumstance. The calculation formula of acoustic radiation force [2] is

$$F_{rad} = -\nabla\left\{\frac{4}{3}\pi R_s^{\,3}\left[(-\frac{3\rho_s - 3\rho_o}{2\rho_s + \rho_o})\cdot\frac{1}{2}\rho_o\langle v\rangle^2 + (1-\frac{\rho_o c_o^{\,2}}{\rho_s c_s^{\,2}})\cdot\frac{1}{2}\frac{\langle p\rangle^2}{\rho_o c_o^{\,2}}\right]\right\} \quad \text{(S2)}$$

where ρ_s is the density of the manipulated particles, and c_0 and c_s are sound speeds in water and in the manipulated particles, respectively. The detailed parameter values of the manipulated particles and water are listed in Table 1. Under the mesh constitution for the PM, the calculated acoustic radiation force distributions for yeast cells and SiNPs at the droplet-substrate interface are depicted in Figures S3a and S3b, respectively. It can be obtained from Figures S3a and S3b that the orders of magnitude of acoustic radiation force at the droplet-substrate interface are 1 fN for yeast cells and 0.01 fN for SiNPs, respectively, which are much smaller than those of the drag force induced by acoustic streaming (100 pN for yeast cells and 10 pN for SiNPs). Therefore, it can be generally concluded that in the ultrasonic probe-droplet-substrate system, acoustic streaming is dominant when the radius of manipulated particles falls between 100 nm and 10 microns.

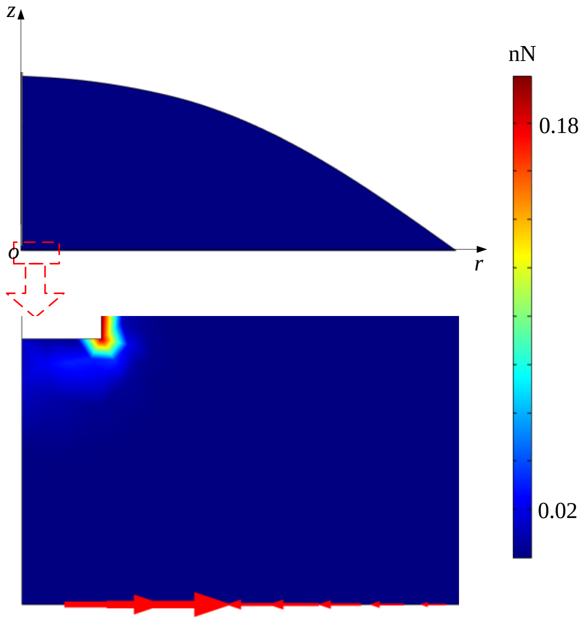

(**a**)

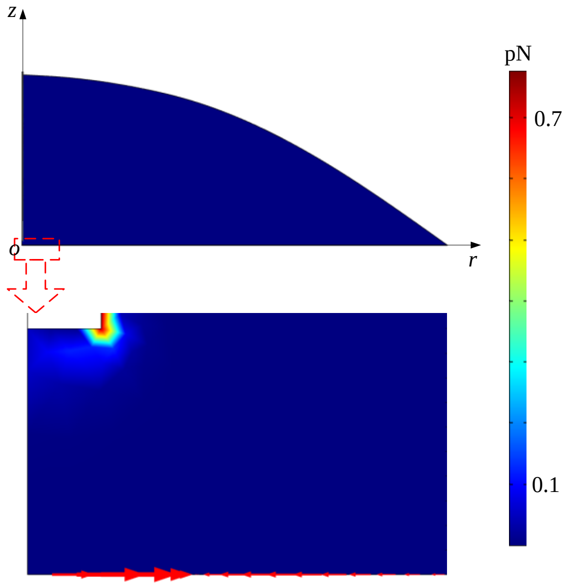

(**b**)

Figure S3. (**a**) Computed acoustic radiation force field for yeast cells in the ultrasonic probe-droplet-substrate system under the mesh constitution for the PM. (**b**) Computed acoustic radiation force field for SiNPs in the ultrasonic probe-droplet-substrate system under the mesh constitution for the PM.

4. Comparison between Acoustic Streaming Induced Drag Force and Acoustic Radiation Force in the Droplet-Ultrasonic Substrate System

In the droplet-ultrasonic substrate system for micro/nanoscale particle concentration, the drag forces along the r axis caused by the r-directional acoustic streaming flow in Figures 10a and 10b, as well as the acoustic radiation force distributions for yeast cells and AgNPs at the droplet-substrate interface under the mesh constitution for the PM, are calculated by Equations (S1) and (S2), respectively. The calculated acoustic radiation force distributions for yeast cells and AgNPs at the droplet-substrate interface are depicted in Figures S4a and S4b, respectively, from which it can be obtained that the orders of magnitude of acoustic radiation force at the droplet-substrate interface are 0.1 fN for yeast cells and 0.001 fN for AgNPs, respectively, which are much smaller than those of

the drag force induced by acoustic streaming (100 pN for yeast cells and 10 pN for AgNPs). Therefore, it can also be generally concluded that in the droplet-ultrasonic substrate system, acoustic streaming is also dominant when the radius of manipulated particles falls between 100 nm and 10 microns.

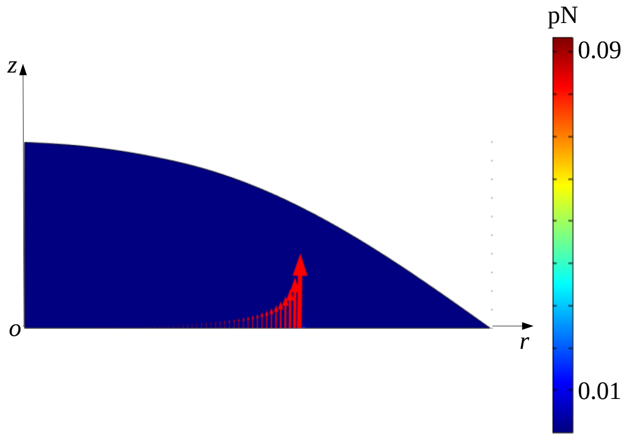

(**a**)

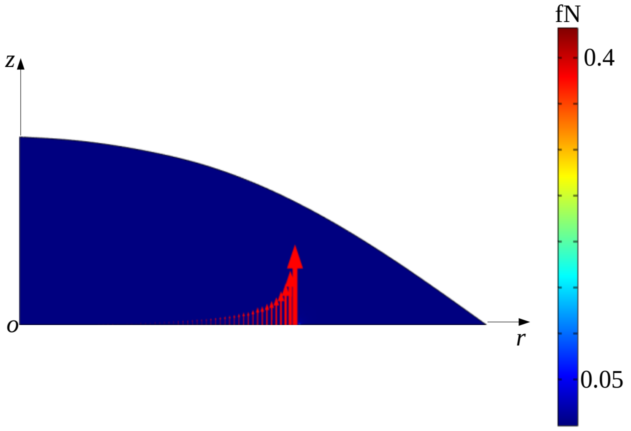

(**b**)

Figure S4. (**a**) Computed acoustic radiation force field for yeast cells in the droplet-ultrasonic substrate system under the mesh constitution for the PM. (**b**) Computed acoustic radiation force field for AgNPs in the droplet-ultrasonic substrate system under the mesh constitution for the PM.

References

1. White, F. M., *Fluid Mechanics*. SEVENTH EDITION ed.; McGraw-Hill: 2010.
2. Gor'kov, L. P., On the Forces Acting on a Small Particle in an Acoustical Field in an Ideal Fluid. *Soviet Physics Doklady* **1962,** 6 (1), 773–775.

© 2019 by the authors. Submitted for possible open access publication under the terms and conditions of the Creative Commons Attribution (CC BY) license (http://creativecommons.org/licenses/by/4.0/).
